# Hypertonic saline- and detergent-accelerated EDTA-based decalcification better preserves mRNA of bones

**DOI:** 10.1038/s41598-024-61459-8

**Published:** 2024-05-13

**Authors:** Zhongmin Li, Clara Wenhart, Andreas Reimann, Yi-Li Cho, Kristin Adler, Goetz Muench

**Affiliations:** Advancecor GmbH, Lochhamerstr. 29 A, 82152 Martinsried, Germany

**Keywords:** Technique, Decalcification, EDTA, Histology, Delipidation, Bone, Biological techniques, Cell biology, Anatomy, Medical research

## Abstract

Ethylenediaminetetraacetic acid (EDTA), a classically used chelating agent of decalcification, maintains good morphological details, but its slow decalcification limits its wider applications. Many procedures have been reported to accelerate EDTA-based decalcification, involving temperature, concentration, sonication, agitation, vacuum, microwave, or combination. However, these procedures, concentrating on purely tissue-outside physical factors to increase the chemical diffusion, do not enable EDTA to exert its full capacity due to tissue intrinsic chemical resistances around the diffusion passage. The resistances, such as tissue inner lipids and electric charges, impede the penetration of EDTA. We hypothesized that delipidation and shielding electric charges would accelerate EDTA-based penetration and the subsequent decalcification. The hypothesis was verified by the observation of speedy penetration of EDTA with additives of detergents and hypertonic saline, testing on tissue-mimicking gels of collagen and adult mouse bones. Using a 26% EDTA mixture with the additives at 45°C, a conventional 7-day decalcification of adult mouse ankle joints could be completed within 24 h while the tissue morphological structure, antigenicity, enzymes, and DNA were well preserved, and mRNA better retained compared to using 15% EDTA at room temperature. The addition of hypertonic saline and detergents to EDTA decalcification is a simple, rapid, and inexpensive method that doesn't disrupt the current histological workflow. This method is equally or even more effective than the currently most used decalcification methods in preserving the morphological details of tissues. It can be highly beneficial for the related community.

## Introduction

Decalcification removes inorganic minerals from the organic matrix of bones, bone-containing specimens, and teeth and is routinely used in laboratories. The softened tissues after decalcification are compatible with routine paraffin-embedding and sectioning for accurate diagnoses in histopathology. The methods used for decalcification are often chosen to provide optimal outcomes for histological stains, immunostaining, in situ hybridization, or molecular tests. The highest quality is indicated by well-preserved tissue structures and cell details, adequate antigenicity, and high confidence in identifying molecular biomarkers or cell nuclear acids. However, the intact morphology of calcified tissue is often difficult to preserve following decalcification with acidic decalcifiers (e.g., formic acid, Hydrochloric acid, etc.) although they are widely used because they provide rapid decalcification. Exposure to the harsh chemicals of acidic decalcifiers is reported to damage the soft tissue structure and negatively affect cellular integrity, antigenicity, and the integrity of DNA^1–3^.

Ethylenediaminetetraacetic acid (EDTA), a classical chelating agent for decalcification, (1) reacts with calcium by binding with the ionized calcium on the outer layer of the apatite crystal, (2) has no effect on the surrounding tissue or tissue depleted of calcium^4,5^, and (3) accordingly provides suitable preservation of the tissue integrity and histological features, enzymes, antigenicity^6–8^, DNA and RNA^2,3,9–11^. The attributes of the EDTA-based decalcification, which are necessary for immunohistochemistry and in situ hybrid analysis, are more important nowadays, as molecular and immunological diagnostics have become part of the standard of care for patients with cancers, such as bone-metastasized or calcified cancers. However, its application has been mostly restricted owing to the slower decalcifying process than acids^5,12^. The time lag can be detrimental to both tissue morphology and antigenicity, can hinder productivity, and can delay diagnostic results in clinical settings^13,14^.

To shorten the time needed for EDTA-based decalcification, many additional treatments have been tested. These include raising the temperature^14,15^, agitation^15–17^, electric field^18^, pulsed electric field^19^, ultrasound^20–28^, high pressures and vacuum^16^, sonication and irradiation of microwave^26,29–33^, high concentration^34^, etc. Theoretically, the outside physical factors mentioned above, which enhance the relevant agent diffusion inside tissue by purely mechanical means, would lead to accelerated decalcification. However, what is ignored in the reports, and maybe more important, is that the agents must reduce the intrinsic chemical resistance to diffusion into the tissues. Tissue resistance due to tissue ionic charges and fat content may retard the diffusion and/or effectiveness of EDTA, thereby prolonging the decalcification process.

To improve the tissue’s internal limitations, in this paper, we propose a novel permeability-enhanced method by the addition of detergents and NaCl to the EDTA-based decalcifier. The detergents are supposed to remove the hydrophobic elements or block lipophilic groups around the ion passages in tissues, and the hypertonic saline might mask the electric charges so that the polar molecules would penetrate the tissues with fewer disturbances, allowing for rapid permeation of the water soluble agent EDTA.

To test the hypothesis, we adopted three strategies: the first was to optimize appropriate concentrations of detergents and saline in a decalcified bone-mimicking substance—gelatin gel, based on penetration rate. To determine whether the optimal agents affect the reaction of EDTA-based decalcification, we performed eggshell experiments. Using the optimal EDTA and additives, the second was to evaluate the decalcification rate in bone tissue, judged by macro-Alizarin stain, under different conditions. To check for feasibility under different important conditions, we repeated the experiment in a varying reagent concentration and temperatures. Finally, the third was to assess the effects on the quality of tissue morphology, antigenicity, and nuclear acids, following decalcification by the new method.

## Results

### EDTA permeability in gelatin gel

**Figure 1:**
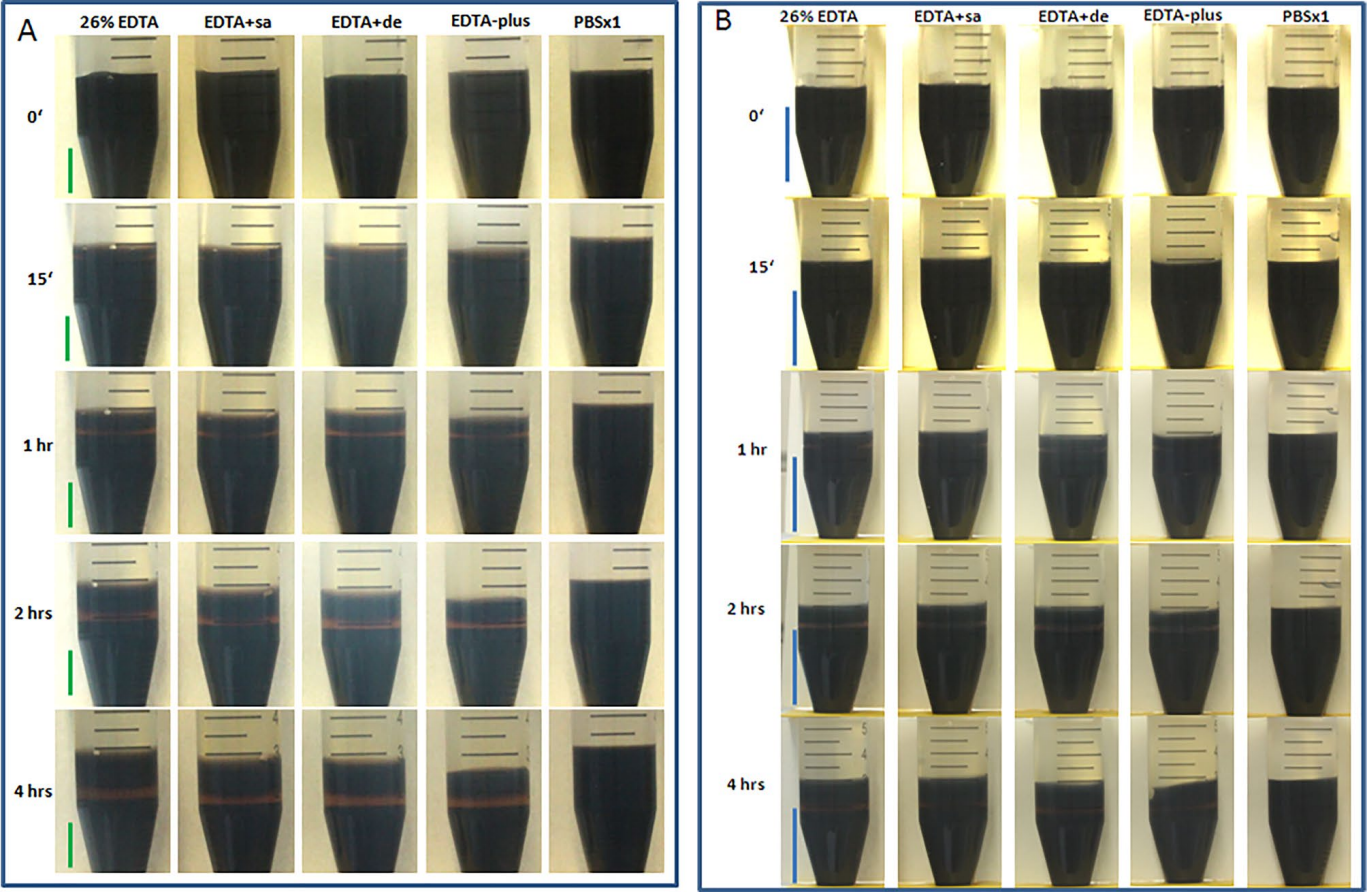

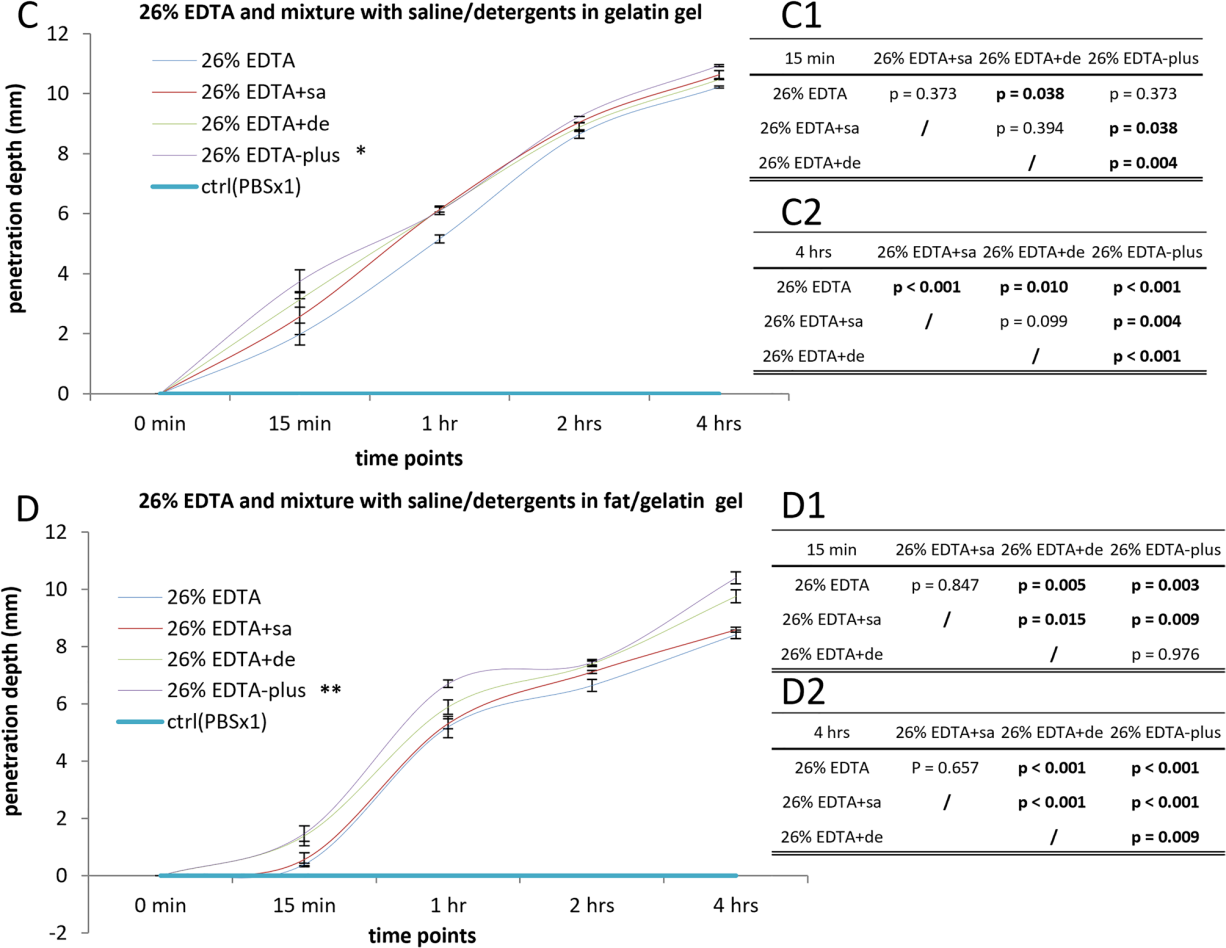
EDTA penetration in gel at different time points (0–4 h). 26% EDTA penetration in the gelatin gel (**A**) or in the fat-containing gelatin gel (**B**) varied with the addition of 5% NaCl (EDTA + sa) or 0.5% Tween/1% triton (EDTA + de), or 5% NaCl and 0.5%Tween/1% triton (EDTA-plus). PBSx1 served as a control. The penetration depth was measured in triplicate at each time point. Comparison of the average values among the experimental groups within the period 15`–4 h, with one-way ANOVA on ranks and Tukey HSD, resulted in a statistically significant difference; *for all, p < 0.002, vs 26% EDTA, 26% EDTA + sa, or 26% EDTA + de; N = 4 (4 average values for 4-time points per group) in the gel without fat (**C**); **for all, p < 0.001, vs. 26% EDTA, 26% EDTA + sa, or 26% EDTA + de; N = 4 in the fat-containing gel (**D**). At the first (15 min) and final (4 h) time points after the addition of EDTA, the penetration depths (N = 3 per group) were compared among the experimental groups, with one-way ANOVA and Tukey HSD. The p values of the pairwise comparisons were given in Tables C1 (15 min) and C2 (4 h) for graph C, and Tables D1 (15 min) and D2 (4 h) for graph (**D**). In the tables statistically significant differences were marked in boldface. Green bars in (**A**) = 10 mm. Blue bars in (**B**) = 20 mm.

Bone mainly consists of collagens and inorganic minerals, and the high permeability of EDTA in the collagens is essential for the speedy decalcification of bone. To estimate the speed of the penetration of EDTA in the collagen of bone, we prepared collagen-rich gelatin gel (w/v, 4%) in diluted Weigert hematoxylin (Weigert A + B, see "Materials and methods" Section) and added EDTA or the mixture solutions on the casted gel in tubes. Prior to and after the addition of the EDTA, the penetration depth was measured at various time intervals including 15 min, 1 h, 2 h, 4 h, and/or 6 h. This was done by measuring the thickness of the light brown color in the gel, as shown in Fig. 1, Figs. S1, S4–S6.

The principle behind the measurements is that the dark-colored Weigert hematoxylin (Weigert A mixed with Weigert B) in the gel became light brown when contacted with EDTA (refer to Fig. S3 for more details).

To optimize the concentration of saline and detergents—the potential enhancers for EDTA-based decalcification, multiple concentrations of NaCl and detergents were prepared with 15% EDTA, and EDTA penetration depths were measured at several time points (see Fig. 1, Figs. S4–S6). Based on the penetration depth, the optimal concentrations for saline and detergents were 5%, and a combination of 0.5% tween-20 and 1% triton-X100, respectively (see Figs. S4, S5), which had the fastest rate of diffusion of EDTA in its group. It should be stated that 10% saline gave out an equal effect to 5% saline (Fig. S4) but was excluded from the list due to being just partially dissolvable during the subsequent preparation of a mixture of highly concentrated detergents. To search for the possibility of additivity of saline-detergent mixture on the EDTA diffusion, in another further experiment (Fig. S6), we prepared a mixture of the optimized concentrations of saline and detergents (5% saline and 0.5%Tween 20/1% Triton X-100 prepared in 15% EDTA, and mixed,15% EDTA-plus) and found that the mix was superior to either the optimized concentrated saline or detergent group alone in the penetration rate.

To test the effects of the additives on the penetration of different concentrations of EDTA, we prepared 26% EDTA (w/v) and its mixtures with the optimized concentrated saline (26% EDTA + sa), detergents (26% EDTA + de), or both (26% EDTA-plus), respectively. We mounted the solutions on the gels, and measurements of EDTA penetration at room temperature were performed at different time points (see Fig. 1A). As a result, we found that 26% EDTA-plus induced the fastest penetration of EDTA among the groups (Fig. 1A,C). The result was further confirmed in repeat experiments and there were statistically significant differences, in comparison of the average values among groups, with one-way ANOVA on ranks followed by Tukey HSD (Tukey's Honestly Significant Difference test) (see Fig. 1C).

From the results, we can speculate that the range of NaCl concentrations above 5% might potentially cancel the ionic interaction of the collagen-mimicking gel, and the mix of 0.5%Tween 20 and 1% Triton X-100 may weaken the action of lipophilic groups of amino acids in the gel. Thus EDTA—the polar molecule would go through the passage of the gel with fewer resistances, leading to a higher penetration rate.

For a study of detergent diffusion into fat-containing tissues, we prepared gelatin gels with the addition of fat extracted from a mouse liver. 26% EDTA and the mixtures mentioned above were added to the gels and the penetration depth was measured at each time point and the analysis is shown in Fig. 1B. The overall outcomes showed that the addition of fat in the gel slowed down the EDTA penetration in EDTA and saline-containing EDTA groups (see Fig. 1B,D) with respect to the EDTA penetration of the same groups in the gel without the addition of fat at each time point (see Fig. 1A,C), suggesting that the fat may retard EDTA diffusion. However, the detergent-containing EDTA, inclusive of 26% EDTA-plus and 26% EDTA + de groups (see Fig. 1B,D), exerted their pronounced effects on penetration in comparison with the gel of non-detergent-contained groups, implying that the detergents may remove the fat and help enhance EDTA diffusion (see Fig. 1B,D).

In conclusion, EDTA containing the additives of hypertonic saline or detergents assists in rapid penetration through the collagen-mimicking gel maybe by masking or delisting the charges or lipophilic elements which hinder the diffusion of EDTA, resulting in high permeability. 26% EDTA-plus exerts the strongest effects on the penetration rate in comparison with the counterparts.

### Hypertonic saline and detergents do not affect the chelate reaction of EDTA

We have shown that both hypertonic saline and detergents can accelerate the penetration of EDTA in tissue-mimicking gels. The next issue that needs to be answered is whether the agents in such a hypertonic concentration affect the reaction of EDTA-based decalcification since normal chelate reactions require a proper microenvironment. To address the question, we prepared powder of chicken eggshell. Chicken eggshell is a composite material containing over 94% calcium salt, and thus is an ideal natural source for studying the decalcification reaction with minimal outside influence, such as diffusion.

The eggshell powder was incubated with 26% EDTA mixtures of hypertonic saline and/or detergent at room temperature (see Fig. S7). 26% EDTA alone and PBSx1 in parallel served as controls. The eggshell powder loss in weight after decalcification (difference of powder weight before and after decalcification incubation) was determined. The results showed a similar weight loss at each time point among the decalcification groups (Fig. S7) and nearly no weight loss among the time points for the PBSx1 group. The repeat experiments reflected a similar outcome and there was no statistically significant difference among the decalcification groups in terms of the average values for each time point. The results indicate that the addition of hypertonic saline and the detergents into the EDTA solution does not interfere with the decalcification reactions.

### Quantification of decalcification in tibia of mouse

**Figure 2:**
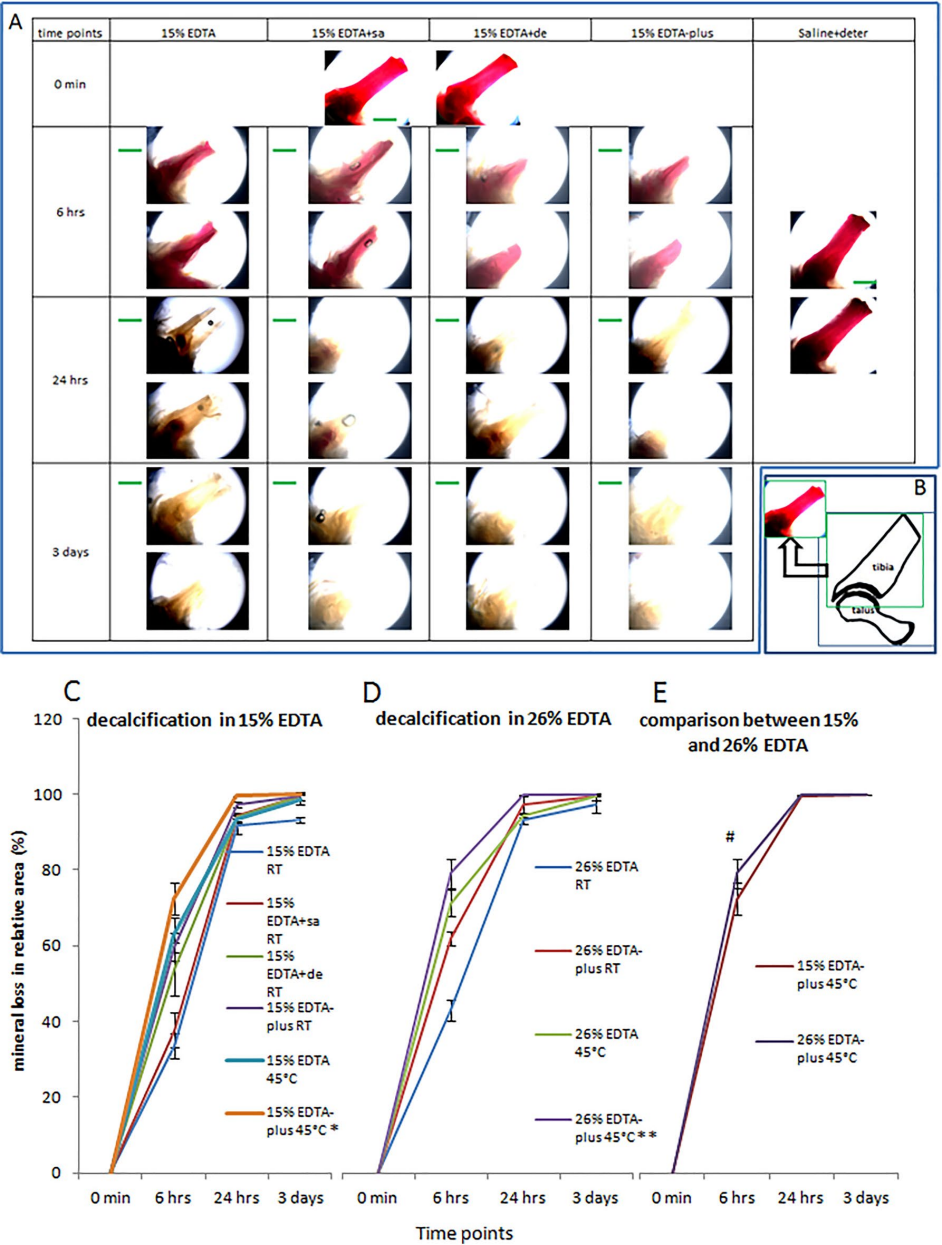
Mineral retention via EDTA decalcification of different methods in the distal portion of tibia. (**A**) The macroscopic stain of Alizarin following decalcification of 15% EDTA, 15% EDTA with additive of 5% saline (15% EDTA + sa), 15% EDTA with additive of detergent mixture of 0.5% tween-20 and 1% triton-X100 (15% EDTA + de), and 15% EDTA with additive of both saline and detergents in the same concentration (15% EDTA-plus) at room temperature (RT, 23 °C). A mixture of 0.5% tween-20, 1% triton-X100 and 5% NaCl (saline + deter) served as a control. The red color indicates the presence of calcium. (**B**) The region of interest (green rectangle) in the distal portion of the tibia was shown as an image (larger green rectangle). (**C**,**D**). The mineral loss in the relative area measured in triplicate or quadruplicate was compared among the groups. The comparison of the average values among the experimental groups within the period 6 h—3 days, with one-way ANOVA on ranks and Fisher LSD test, resulted in a statistically significant difference; *for all, p < 0.04, vs. 15% EDTA RT, 15% EDTA + sa RT, 15% EDTA + de RT, 15% EDTA-plus RT, or 15% EDTA 45 °C; N = 3 (3 average values for 3-time points per group) in (**C**); **for all, p < 0.001, vs 26% EDTA RT, 26% EDTA-plus RT, or 26% EDTA 45 °C; N = 3 in (**D**). (**E**) Comparison between the values of 15% EDTA-plus and 26% EDTA-plus at a higher temperature (45°C), measured at 6 h, with Paired Samples T-tests, led to a significant statistical difference; #, p < 0.01 of 2-tailed, N = 4. Green bars in (**A**) = 1 mm.

To evaluate the efficiency of the addition of hypertonic saline and detergents on EDTA decalcification of bone tissue, a group of mouse hind paws of similar weight in the same age, species, and sex (see Table S1) was skinned, fixed, and randomly submersed in solutions of 15% EDTA, 15% EDTA + sa (5% saline prepared in 15% EDTA), 15% EDTA + de (1% triton (v/v) and 0.5% tween (v/v) prepared in 15% EDTA), and 15% EDTA-plus (5% saline, 1% triton, and 0.5% tween prepared in 15% EDTA) for decalcification, and in PBSx1 for a control. Prior to and after being incubated at room temperature under continuous agitation for 6 h, 24 h, and 3 days, the specimens were collected and subjected to Alizarin staining. The mineral content of the tibia in the distal portion, indicated by the Alizarin-stained red color (see Fig. 2A,B), was measured by image analysis. The mineral retaining in the relative area is the ratio between the mineral content area of red captured in a relatively long exposure light image and the sample contour area displayed in a relatively short exposure light image. The mineral loss in the relative area via decalcification was the value of the retained mineral in the relative area subtracted from 100%. The outcomes showed that the mineral loss via the decalcification with EDTA mixture either of saline or detergents or with saline and detergents is above EDTA used alone at each time point, and 15% EDTA-plus was proved to be the most efficient solution in decalcification in comparison with those of other counterpart solutions at a temperature (room temperature, RT or 45 °C) (see Fig. 2C). As a control (PBSx1), the mineral content was kept unchanged. The subsequent repeated experiments demonstrated a similar result. Comparisons of average values among the groups, with one-way ANOVA on ranks, or comparisons of single values at each time point, with one-way ANOVA, followed by Fisher LSD test (Fisher’s protected Least Significant Difference Test), resulted in statistically significant differences (see Fig. 2C and Table 1).

**Table 1:** Percent of mineral loss (%) (means ± SD) and p values following pairwise comparisons at different time points.

6 h		15% EDTA RT	15% EDTA + sa RT	15% EDTA + de RT	15% EDTA-plus RT	15% EDTA 45 °C	15% EDTA-plus 45 °C	26% EDTA RT	26% EDTA-plus RT	26% EDTA 45 °C	26% EDTA-plus 45 °C
		33.65 ± 3.26	37.74 ± 4.52	53.81 ± 7.07	59.75 ± 3.81	62.80 ± 4.53	72.33 ± 4.19	42.97 ± 2.63	61.91 ± 1.86	71.25 ± 3.47	79.09 ± 3.81
15% EDTA RT	33.65 ± 3.26	**/**	p = 0.229	**p < 0.001**	**p < 0.001**	**p < 0.001**	**p < 0.001**	**p = 0.01**	**p < 0.001**	**p < 0.001**	**p < 0.001**
15% EDTA + sa RT	37.74 ± 4.52		**/**	**p < 0.001**	**p < 0.001**	**p < 0.001**	**p < 0.001**	p = 0.128	**p < 0.001**	**p < 0.001**	**p < 0.001**
15% EDTA + de RT	53.81 ± 7.07			**/**	p = 0.086	**p = 0.013**	**p < 0.001**	**p = 0.003**	**p = 0.023**	**p < 0.001**	**p < 0.001**
15% EDTA-plus RT	59.75 ± 3.81				**/**	p = 0.366	**p < 0.001**	**p < 0.001**	p = 0.52	**p = 0.002**	**p < 0.001**
15% EDTA 45 °C	62.80 ± 4.53					**/**	**p = 0.003**	**p < 0.001**	p = 0.791	**p = 0.018**	**p < 0.001**
15% EDTA-plus 45 °C	72.33 ± 4.19						**/**	**p < 0.001**	**p = 0.002**	p = 0.583	**p = 0.044**
26% EDTA RT	42.97 ± 2.63							**/**	**p < 0.001**	**p < 0.001**	**p < 0.001**
26% EDTA-plus RT	61.91 ± 1.86								**/**	**p = 0.01**	**p < 0.001**
26% EDTA 45 °C	71.25 ± 3.47									**/**	**p = 0.019**
26% EDTA-plus 45 °C	79.09 ± 3.81										**/**

24 h		15% EDTA RT	15% EDTA + sa RT	15% EDTA + de RT	15% EDTA-plus RT	15% EDTA 45 °C	15% EDTA-plus 45 °C	26% EDTA RT	26% EDTA-plus RT	26% EDTA 45 °C	26% EDTA-plus 45 °C
		91.60 ± 2.03	94.19 ± 0.67	93.85 ± 1.06	97.30 ± 0.78	93.51 ± 0.94	99.56 ± 0.02	93.39 ± 1.40	97.43 ± 2.24	94.48 ± 0.53	100 ± 0
15% EDTA RT	91.60 ± 2.03	**/**	**p = 0.016**	**p = 0.033**	**p < 0.001**	p = 0.066	**p < 0.001**	p = 0.083	**p < 0.001**	**p = 0.008**	**p < 0.001**
15% EDTA + sa RT	94.19 ± 0.67		**/**	p = 0.734	**p = 0.005**	p = 0.495	**p < 0.001**	p = 0.424	**p = 0.003**	p = 0.771	**p < 0.001**
15% EDTA + de RT	93.85 ± 1.06			**/**	**p = 0.002**	p = 0.729	**p < 0.001**	p = 0.643	**p = 0.002**	p = 0.53	**p < 0.001**
15% EDTA-plus RT	97.30 ± 0.78				**/**	**p < 0.001**	**p = 0.032**	**p < 0.001**	p = 0.889	**p = 0.009**	**p = 0.012**
15% EDTA 45 °C	93.51 ± 0.94					**/**	**p < 0.001**	p = 0.905	**p < 0.001**	p = 0.334	**p < 0.001**
15% EDTA-plus 45 °C	99.56 ± 0.02						**/**	**p < 0.001**	**p = 0.042**	**p < 0.001**	p = 0.66
26% EDTA RT	93.39 ± 1.40							**/**	**p < 0.001**	p = 0.28	**p < 0.001**
26% EDTA-plus RT	97.43 ± 2.24								**/**	**p = 0.007**	**p = 0.016**
26% EDTA 45 °C	94.48 ± 0.53									**/**	**p < 0.001**
26% EDTA-plus 45 °C	100 ± 0										**/**

3 days		15% EDTA RT	15% EDTA + sa RT	15% EDTA + de RT	15% EDTA-plus RT	15% EDTA 45 °C	15% EDTA-plus 45 °C	26% EDTA RT	26% EDTA-plus RT	26% EDTA 45 °C	26% EDTA-plus 45 °C
		93.25 ± 0.57	99.36 ± 1.10	99.37 ± 1.10	99.44 ± 0.97	98.64 ± 1.18	100 ± 0	97.41 ± 2.25	99.44 ± 0.96	99.46 ± 0.94	100 ± 0
15% EDTA RT	93.25 ± 0.57	/	**p < 0.001**	**p < 0.001**	**p < 0.001**	**p < 0.001**	**p < 0.001**	**p < 0.001**	**p < 0.001**	**p < 0.001**	**p < 0.001**
15% EDTA + sa RT	99.36 ± 1.10		/	p = 0.998	p = 0.932	p = 0.427	p = 0.483	**p = 0.041**	p = 0.928	p = 0.915	p = 0.483
15% EDTA + de RT	99.37 ± 1.10			/	p = 0.934	p = 0.425	p = 0.485	**p = 0.041**	p = 0.931	p = 0.917	p = 0.485
15% EDTA-plus RT	99.44 ± 0.97				/	p = 0.38	p = 0.537	**p = 0.034**	p = 0.997	p = 0.983	p = 0.537
15% EDTA 45 °C	98.64 ± 1.18					/	p = 0.143	p = 0.184	p = 0.378	p = 0.369	p = 0.143
15% EDTA-plus 45 °C	100 ± 0						/	**p = 0.009**	p = 0.54	p = 0.552	p = 1
26% EDTA RT	97.41 ± 2.25							/	**p = 0.034**	**p = 0.033**	**p = 0.009**
26% EDTA-plus RT	99.44 ± 0.96								/	p = 0.986	p = 0.54
26% EDTA 45 °C	99.46 ± 0.94									/	p = 0.552
26% EDTA-plus 45 °C	100 ± 0										/

The mineral loss in the relative area via decalcification of different methods, measured in triplicate or quadruplicate (N = 3 or 4) was compared. The comparison within the period 6 h–3 days, with one-way ANOVA and Fisher LSD test, resulted in different p values. A p < 0.05 was considered statistical significance. Statistically significant differences were marked in boldface.

To test the feasibility of the application under different conditions, we focused on the decalcification efficiency of using a higher EDTA concentration of 26%, and of raising temperature to 45 °C (Fig. 2D). Similar results to the use of 15% EDTA (Fig. 2C) were observed (Fig. 2D). A significant difference was found at 6 h with 26% EDTA-plus 45°C giving a more rapid decalcification rate (Fig. 2D, Table 1).

The results demonstrated that a higher temperature of decalcification or a higher concentration of EDTA led to a greater efficiency of decalcification, compared to the other combinations tested (Fig. 2C–E). When the optimal conditions found in Fig. 2C,D were compared directly (Fig. 2E), then the 26% EDTA-plus 45°C was significantly higher than the 15% EDTA-plus 45 °C at 6 h. 26% EDTA-plus at 45°C led to complete decalcification (100%) within 24 h but 15% EDTA-plus to 99.56% at the same temperature (Fig. 2E). The results suggest that the addition of saline or/and detergents can enhance the efficiency of EDTA decalcification and significantly shorten the decalcification time in bone tissue.

### Histological analysis of retention of tissue structure and cellular detail after decalcification

Morphological preservation after decalcification is a prerequisite for the application of the relevant stains. To inspect the potential impacts of a divergent fashion of decalcification on morphological retention, we prepared the right hind paws of a cohort of 12 male mice of a similar age, and body and hind paw weight (see Table S1). 10–15% EDTA at room temperature for decalcification is considered the “gold standard”—which is the technique most cited in the literature. Thus, the samples were randomly and equally divided into two groups. Six paws in one group objected to decalcification with 26% EDTA-plus at 45 °C under agitation for 24 h (26% EDTA-plus)—the most efficient decalcification method verified in our previous tests, and the rest 6 paws in the other group with 15% EDTA at room temperature under agitation (15% EDTA) for one week^35^. Then the samples underwent paraffin embedding before the sagittal sections of ankle joints were cut (Fig. S2).

To test the retention of morphological details following the decalcification, we performed (1) Hematoxylin and eosin (HE) for examination of general tissue structures and cellular details; (2) Safranin O (Saf O) and Toluidin Blue (TB) for inspection of proteoglycan status; and (3) Sirius Red (SR) for evaluation of collagen retention. The stains were performed in the middle sagittal sections of the ankle joints (Fig. S2). The outcomes of the stains of HE, Saf O, and TB were analyzed, with emphasis on the subperichondrial layer of distal articular cartilage of the tibia. The intensity of collagen stain was evaluated in the tibial cortical bone of the distal part (exclusive of bone marrow).

#### HE staining

**Figure 3:**
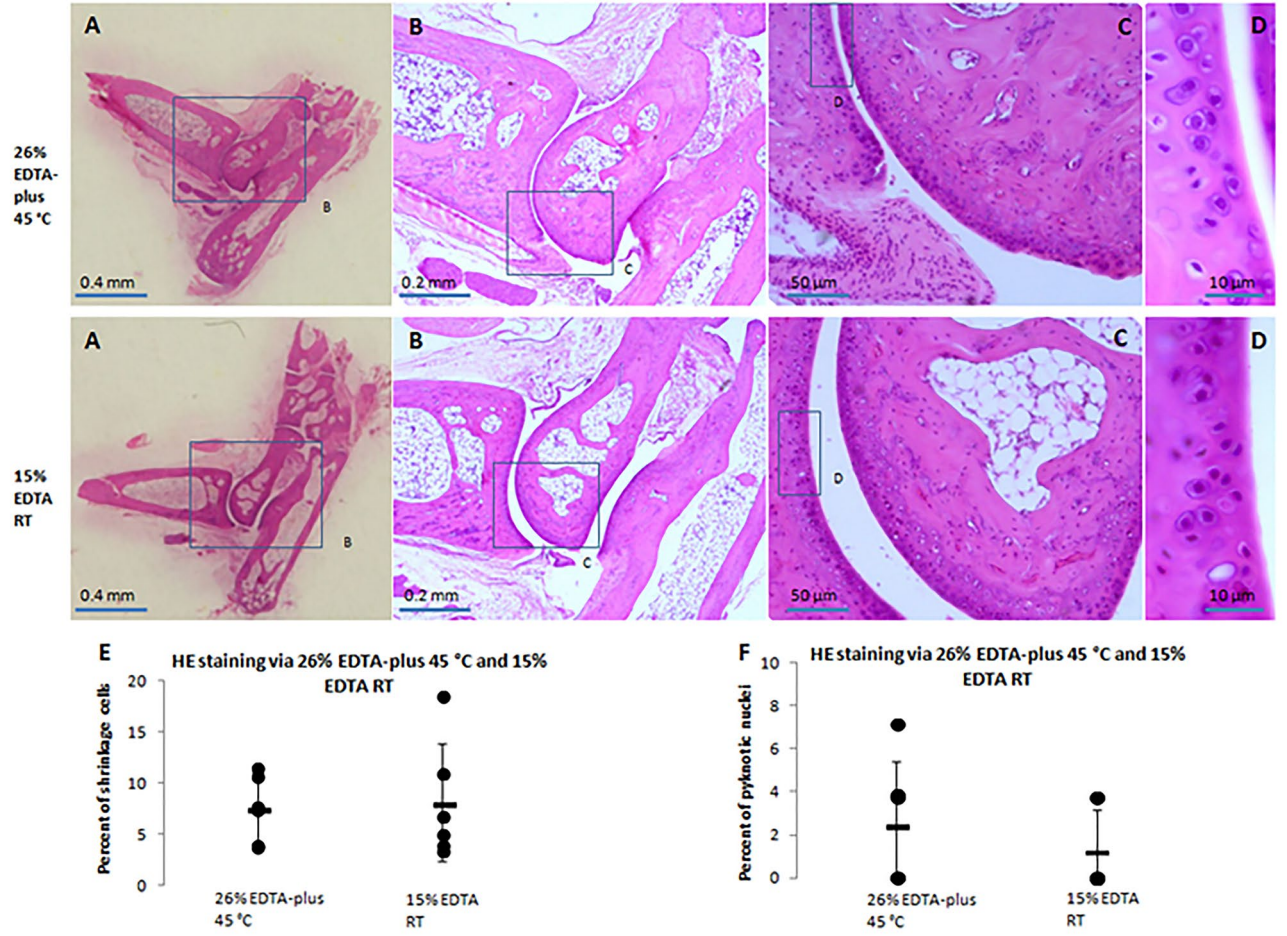
HE stained sections of ankle joints via decalcification of 26% EDTA-plus 45 °C and 15% EDTA RT. The regions indicated by blue rectangles in (**A**–**C**) were magnified in the images of (**B**–**D**), respectively. In terms of the relative shrinkage cells (%) in (**E**) and pyknotic nuclei (%) in (**F**), the comparison between 26% EDTA-plus 45 °C and 15% EDTA RT, with Independent Samples T-test, resulted in statistically non-significant differences (N = 6 per group; p = 0.54 of 2-tailed, for (**E**); P = 0.83 of 2-tailed, for (**F**)).

HE-stained sections were observed using light microscopy. The observations showed that qualified sections, via 26% EDTA-plus (45 °C) decalcification, were well-preserved (see Fig. 3A–D). There were no rips or tears in the sections. The specimen structures and components were kept intact, and the cell structures were clear in contrast with a clean background. No artifacts or detrimental structures were observed. The tissue structures and cellular details via 26% EDTA-plus (45 °C) were like those following the standard technique (see Fig. 3A–D).

For evaluation of cell shrinkage or pyknotic nuclei, which were likely to be produced at a higher temperature and in a hypertonic decalcification solution of 26% EDTA-plus (45 °C), we counted the positive cells or nuclei and all the cells or nuclei examined under a higher magnification (an × 40 objective lens). Light microscopy revealed no qualitative differences in comparison with the standard technique regarding cell shrinkage or pyknotic nuclei (%) and there were no statistically significant differences in comparisons with Independent Samples T-test (Fig. 3E,F).

Pyknosis is defined as the “shrinkage of the nuclear material of a cell into a homogenous hyperchromatic mass”^16^. Shrinkage is defined as the separation between the chondrocyte membrane and the lacunar rim^16^. The shrinkageor pyknotic rate is the ratio of the positive cell or nucleus number to the total number counted in the distal articular cartilage of the tibia.

#### Saf O or TB staining

The stains are often used for the determination of proteoglycan loss and cartilage erosion in inflammatory arthritis. A reliable decalcification should not affect the stains of Saf O or TB. To evaluate the potential effects of the new decalcification method on the proteoglycan stains, we measured the cartilage thickness in the distal end of the tibia, following the stains, and made a comparison with the outcomes via the standard technique (see Figs. S8, S9). The cartilage thickness for each joint was determined by the cartilage area divided by the cartilage surface length. A similar thickness was displayed to that via the standard technique. No staining difference was observed in histological sections between the two groups, suggesting that 26% EDTA-plus (45 °C) did not induce loss of proteoglycan in the tissue.

#### Sirius red staining

The staining is one of the best-understood techniques of collagen histochemistry. The collagen loss is reflected in the staining intensity. We measured the stained orange intensity in the tibial cortical bone with Image Analysis. The results showed that the staining intensity was similar between the two groups, suggesting EDTA-plus did not cause loss of collagen in the tissue (refer to Fig. S10). In the high magnification of the staining, we also found a similar collagen distribution.

### Histochemical analysis of retention of enzyme activity after decalcification

Tartrate-resistant acid phosphatase (TRAP) staining is widely employed for the detection of multinucleated osteoclasts and scoring the extent of bone erosions. To determine whether the new decalcification technique would also accelerate quenching of TRAP enzyme activity, we performed the staining in sections adjacent to those stained with HE, Saf O, TB, or SR. The TRAP staining positive relative area was calculated by the ratio between the positive staining area and the tibia-involved area in the distal shaft region of the tibia. Comparison of the relative area, with Independent Samples T-test, in the tissues via 26% EDTA-plus (45 °C) and 15% EDTA (RT) led to no statistically significant difference (Fig. S11). The results suggest that the new decalcification technique did not affect enzymatic activities.

### Immunohistochemistry analysis of retention of tissue antigenicity after decalcification

Tissue antigenicity including the specificity and sensitivity should not be affected by the processing of the new decalcification style. To evaluate the tissue antigenicity, we carried out immunostaining for inflammation-relevant cells via the new decalcification model. Inflammation is the most common sign in joint diseases and immunological detection for inflammatory cells is often used for evaluation of the severity of the diseases in the laboratory. To develop arthritis, male DBA/1 mice, 8 weeks in age, were immunized and boosted with bovine collagen II and human fibrinogen as previously reported^36^. After 12 weeks of immunization, the left hind paws, which weighed over 0.180 g (0.144–0.151 g of the native mice at the corresponding age, see Table S1), were randomly objected to decalcification either with 15% EDTA (RT) for 1 week or 26% EDTA-plus (45 °C) for 24 h as described. Following the decalcification, we carried out sectioning and HE staining as previously described. The adjacent sections of HE stained which displayed moderate inflammation, were selected for traditional ABC immunohistochemistry (IHC) for Macrophages, CD45, and CD3. To minimize bias due to various sites of observation, we focused on the pannus formation of arthritis in the ankle joints, limiting to invaded portions within the articular cavity.

**Figure 4:**
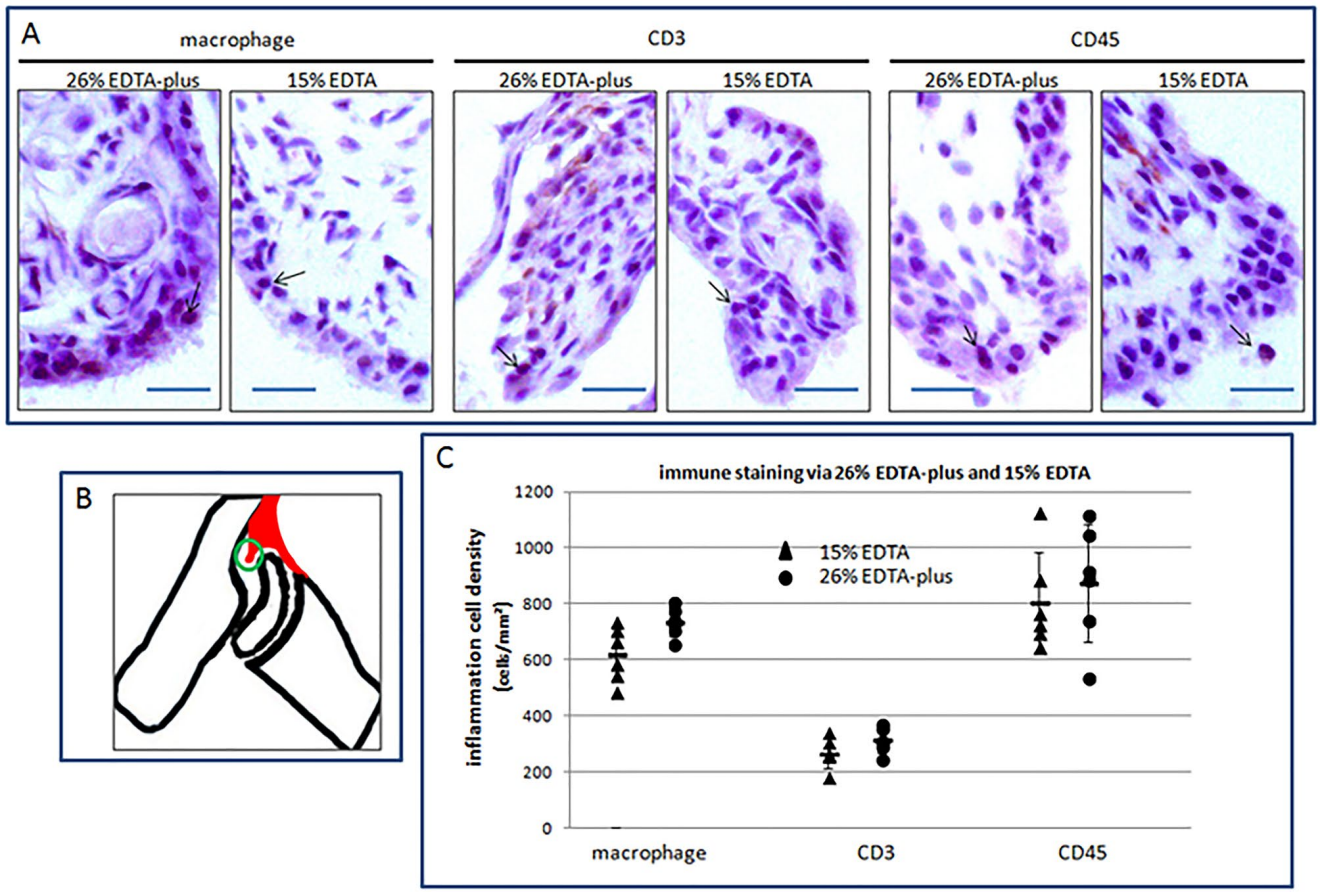
Immunohistochemistry of sections of ankle joints following decalcification of 26% EDTA-plus (45°C) and 15% EDTA (RT). (**A**) Images of immunohistochemistry of ABC for macrophages, CD3, and CD45 were performed with a counterstain of hematoxylin. The positive cells (indicated by arrows) are colorized dark brown. Bars = 20 µm. (**B**) The region of interest (green circle) is localized in the pannus formation of arthritis. The thickened synovium and pannus formation is marked with red. (**C**) Inflammation cell density within the pannus of arthritis via decalcification of 26% EDTA-plus (45 °C) and 15% EDTA (RT) was demonstrated. In terms of the cell density, the comparison between them, with Independent Samples T-test, resulted in statistically non-significant differences (N = 5–6 per group; p = 0.07 of 2-tailed for macrophages; p = 0.13 of 2-tailed for CD3; p = 0.82 of 2-tailed, for CD45).

No staining difference was observed in the histological sections via the new decalcification method, in comparison with corresponding staining via the standard technique, with respect to the targeted cell density (see Fig. 4A,B). For evaluation of the cell density, we first defined the region of the pannus—the invasive part of thickened synovium into the articular cavity and measured the area of the region with Image analysis (see Fig. 4B). In the region, we counted the positive cells in terms of blue nuclei. The positive cell density is the ratio of the positive cell number to the involved area as reported^42,43^. The experiments were validated by the findings of a parallel experiment on the spleen. As positive controls for all the targets, the spleen sections revealed positive, and blank controls, without application of the relevant antibodies, displayed negative.

### Nuclear staining and in situ hybrid analysis of retention of cellar DNA

Good retention of cellular nuclear acid after decalcification is very important for current molecular pathology. To evaluate the retention of quantity and quality, we did HE and DAPI fluorescence staining, and whole chromosome Y painting in the decalcified ankle joints with either 26% EDTA-plus (45 °C) or 15% EDTA (RT) as described previously. DAPI fluorescence staining and the chromosome Y painting were performed in the adjacent sections of HE stained. The joints were derived from a cohort of mice in a similar background (see Table S1).

**Figure 5:**
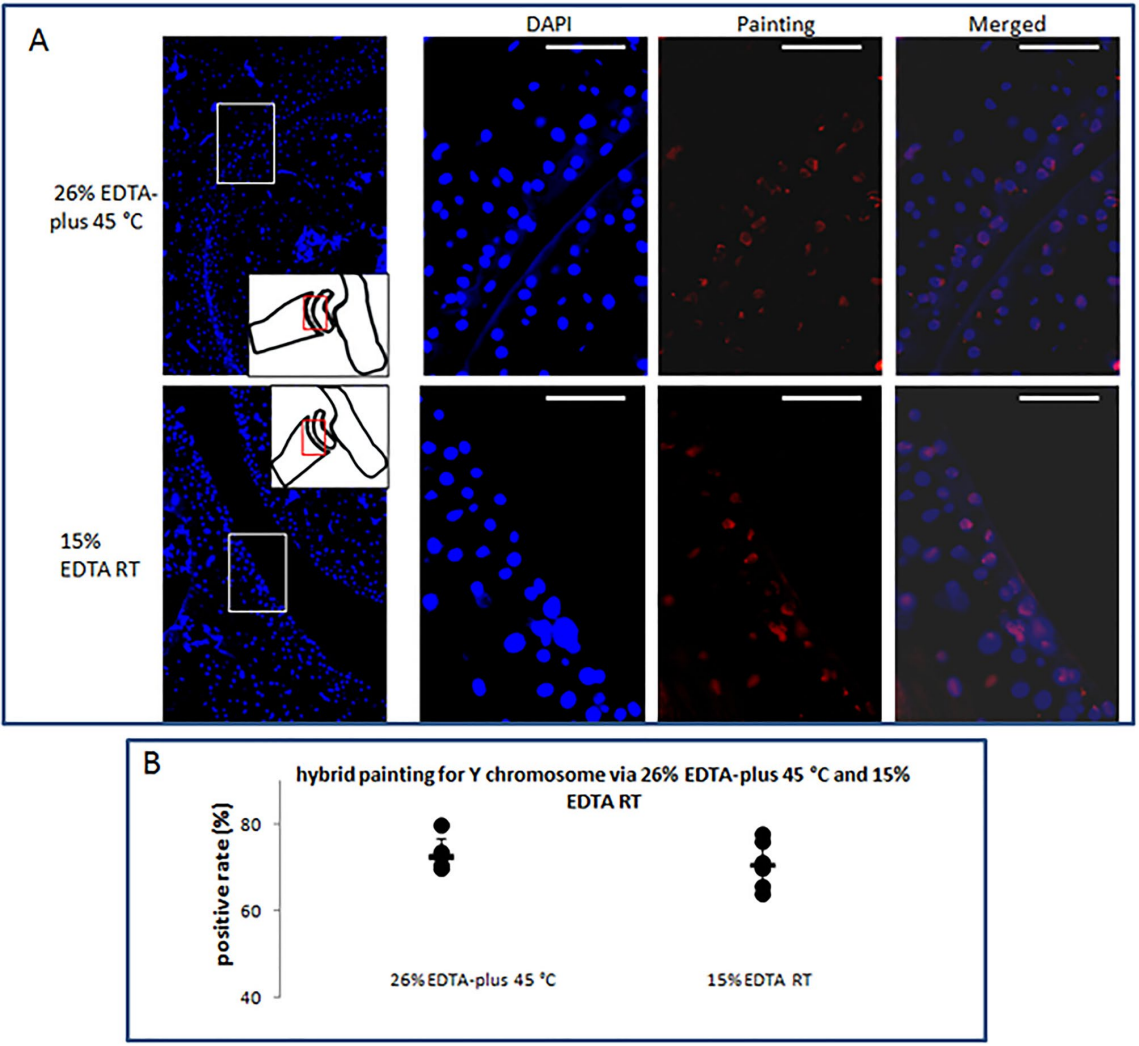
DAPI staining and in situ hybrid painting for Y chromosome of moue. (**A**) Images of the staining. The regions indicated by red rectangles in insets were magnified in the corresponding images and the regions of interest (white rectangles) were observed under DAPI and Cy-3 channels. Bar = 50 µm. (**B**) Y chromosome painting positive rates were calculated and compared in the tissues decalcified with 26% EDTA-plus 45 °C and 15% EDTA RT. The comparison between the two decalcification fashions, with Independent Samples T-test, resulted in a statistically non-significant difference (N = 6 per group, p = 0.33).

We observed nuclear details of blue color in HE stains (Fig. 3D) and those of blue fluorescence in DAPI staining (Fig. S12) under an × 40 objective lens. The stains looked similar in the tissues via both decalcification styles (Fig. 3D, Fig. S12A). Comparison of the nuclear area per cell in DAPI staining resulted in no statistically significant differences (Fig. S12B). To assess nuclear acid integrity, a mouse Whole Chromosome Y Painting FISH Probe was used to determine the copy number and integrity of complete mouse chromosome Y and detect the possibility of abnormalities of the chromosome. The painting was completed with counterstaining of DAPI (see Fig. 5A). Blending the nuclear-stained blue and the chromosome Y-stained red fluorescence allowed for the identification of positive nuclei. We counted the positive nuclei of both red and blue, and the total nuclei of blue. The positive proportion to the total (positive rate) was calculated. Comparison of the positive rate resulted in a high closeness in the tissues via both decalcification styles (no significant statistical difference between them, see Fig. 5B).

### RNAscope technology for detection of preservation of gene expression (RNA)

**Figure 6:**
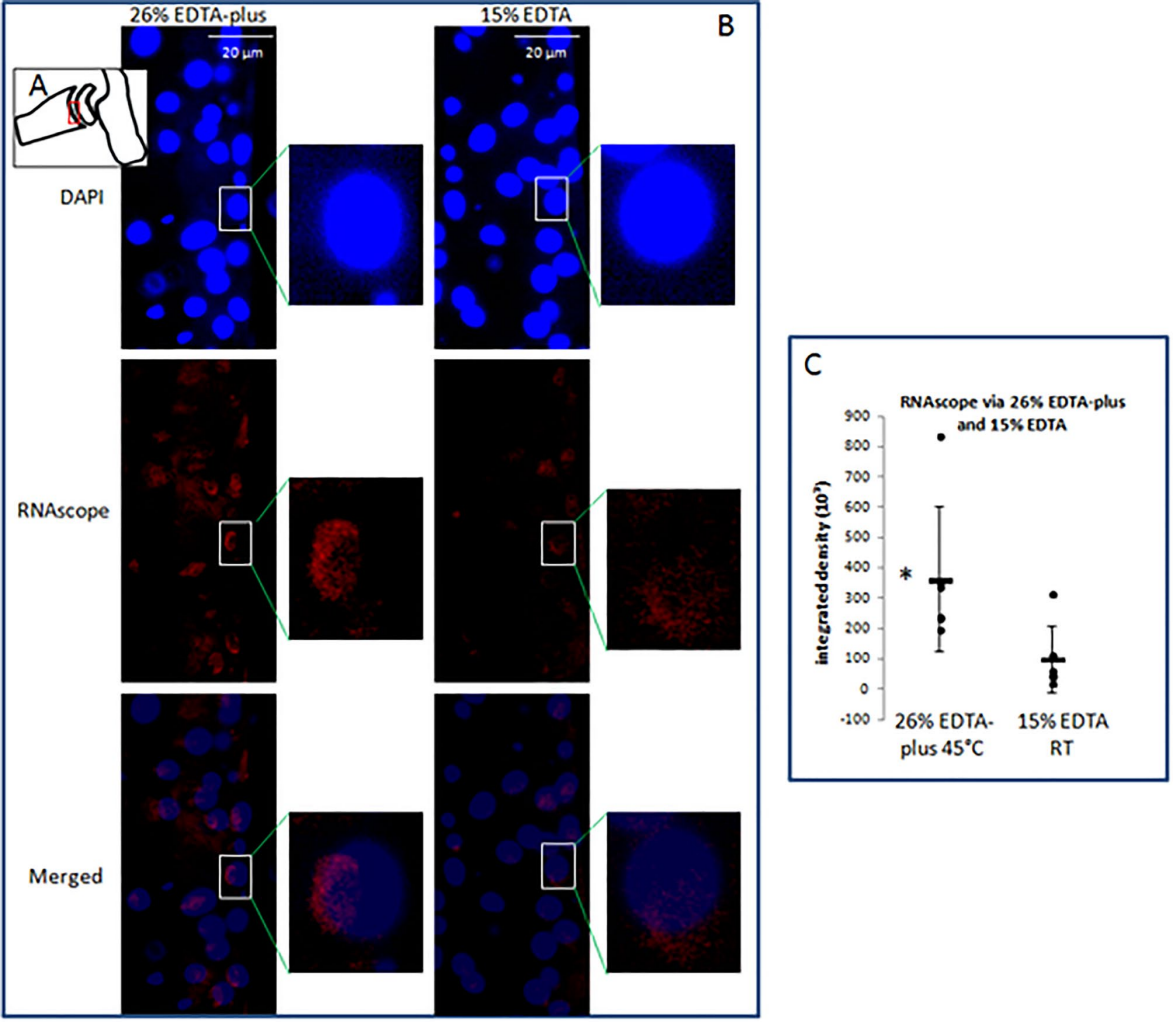
Images of RNAscope and DAPI staining in chondrocytes of mice. The region indicated by the red rectangle in (**A**) was observed in images (**B**) under DAPI and Cy-3 channels. The regions of interest (white rectangles) in (**B**) were magnified in the adjacent corresponding images. Mouse ß-actin mRNA expression in chondrocyte (red fluorescence) was calculated in terms of integrated density and compared in the tissues decalcified with 26% EDTA-plus (45°C) and 15% EDTA (RT). The comparison (**C**) between the two decalcification fashions in the regions limited to the middle portion (100 µm) demarcated as the red rectangle of (**B**), with Independent Samples T-test, resulted in a statistically significant difference (*vs. 15% EDTA RT, N = 6 per group, p = 0.034).

Retention of RNA in decalcified tissue is critical for gene expression analysis in modern molecular biology. RNAscope is a novel and sensitive technology that can be used to measure single RNA molecules per cell in samples mounted on slides. To assess the retention of RNA, an RNAscope was employed and performed in the adjacent sections of HE stained. We selected beta-actin housekeeping genes as a preservation indicator of mRNA since housekeeping genes are commonly described as stably expressed irrespective of tissue type, developmental stage, cell cycle state, or external signal. We observed the main parts of red labeled dots located within one side of the cytoplasm and minor parts within nuclei (see Fig. 6B). Each dot of red fluorescence represents one RNA molecule. However, it is noteworthy that in the case of the highly expressed housekeeping gene – ß-Actin, the dots were found in clusters, which makes them difficult to distinguish separately. We measured the integrated density in the middle chondrocyte region (Fig. 6A) of the distal end of the tibia and compared the density of the samples via the two decalcification styles. Comparisons led to a statistically significant difference (see Fig. 6C), which indicates that the decalcified samples via 26% EDTA-plus (45 °C) better preserve mRNA than those via 15% EDTA (RT) and the difference may be due to a shortened decalcification period of 26% EDTA-plus (45 °C).

### Accuracy and comparison of staining outcomes via 26% EDTA-plus and 15% EDTA

**Figure 7:**
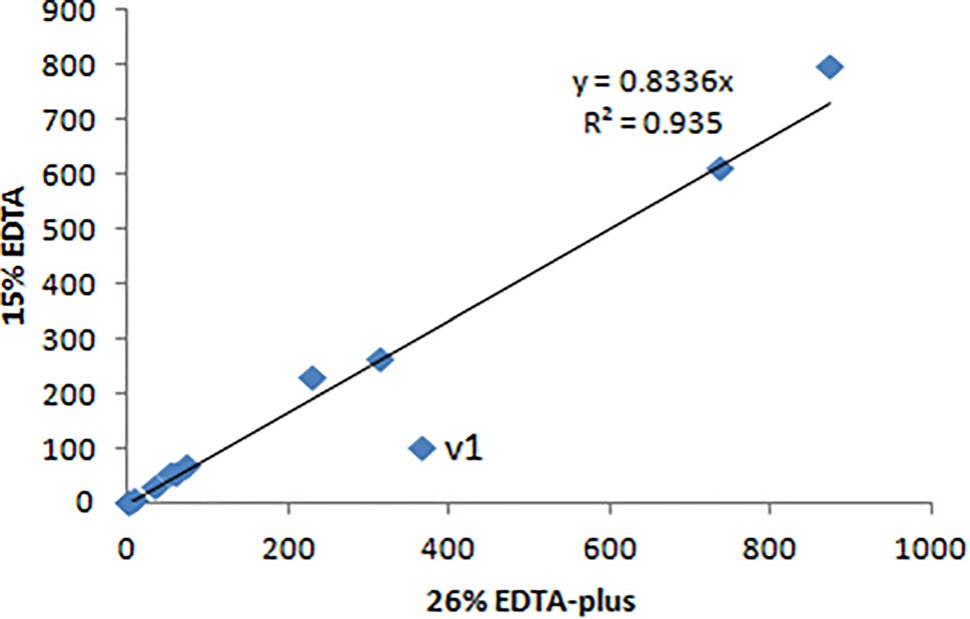
Accuracy testing on average values of each stain in tissues via decalcifications of 26% EDTA-plus (45 °C) and 15% EDTA (RT). The statistical analysis resulted in a statistically significant correlation (N = 12 pairs, Pearson’s r = 0.967, p < 0.001). V1 indicates the data point of RNAscope analysis.

Accuracy is the closeness of agreement between the tested results and accepted reference values^37,38^. Staining results with decalcification of 10–15% EDTA are widely used and accepted for morphological analysis. The degree of closeness between the outcomes via 26% EDTA-plus (45 °C) and 15% EDTA (RT) was tested by assessing the correlation. We pooled the average values determined for each kind of stain in the decalcified tissues with either 26% EDTA-plus (45 °C) or 15% EDTA (RT). The values were from 1. Cell shrinkage rate, 2. Nuclear pyknotic rate, 3. Macrophage, 4. CD3, 5. CD45, 6. DNA painting, 7. DAPI staining, 8. TRAP staining, 9. Saf O staining, 10. TB staining, 11. Sirius staining, and 12. RNAscope. Comparison of the outcomes resulted in a statistically significant correlation (see Fig. 7). The similarity in the morphological features generated via 26% EDTA-plus (45°C) to those with 15% EDTA (RT) confirms that staining outcomes with 26% EDTA-plus (45 °C) are accurate. Of note, an outlier (v1 in Fig. 7), located far away from the trendline (others), is from the corresponding average values of RNAscope. These results further partially confirm that the general relationship in the other categories becomes strong although decalcification via the new method yields more mRNA (discrepancy).

In conclusions: decalcification with 26% EDTA-plus (26% EDTA with the addition of saline and detergents at 45 °C) is considerably accelerated and the speedy decalcification may act through a quick diffusion system by blocking and/or removing tissue intrinsic charges or fat. Thereafter 26% EDTA-plus (45 °C) provides appropriate results for histostaining, histochemistry, immunohistochemistry, and in situ hybrid painting, and better outcomes for mRNA preservation, in comparison with the standard technique. The results imply that 26% EDTA-plus (45 °C) is a valuable and quick decalcified method and may be recommended for urgent needs in calcified tissues.

## Discussion

Chelating agents such as EDTA work by capturing the calcium ions from the outer layer of the apatite crystal at the initial stage, and then the decalcification reaction occurs slowly in the interior of bone tissue. The reaction interface is originally on the surface of the tissue block, and with the advance of decalcification, the interface decreases in size. When decalcification is complete the reaction surface approaches zero. During the decalcification, the thickness of the organic layer (mainly collagen) of decalcified material between the reaction interface and the decalcifier increases, accumulatively impeding diffusion, namely, the interchange of a free supply and removal of the substances involved. The rate of decalcification (amount of calcium salts extracted per unit of time), therefore, will reduce with the development of the process. This is why the rate of decalcification accelerated very quickly at the beginning, but eventually slowed down considerably, particularly in the period of nearly 100% decalcification.

To accelerate the decalcification process, two factors can be manipulated, namely, by increasing the rate of reaction and by increasing the rate of diffusion, respectively. For the rate of reaction, the decalcifying agent—EDTA, and the target tissue—bone have been designated in the case, and accordingly, we can do nothing to interfere with this factor. This has been partially confirmed by the tests on the chicken eggshell (see Fig. S7). However, the diffusion factor can be made to use in any attempt to influence the rate of decalcification. Diffusion is the passive movement of substances from a region of higher concentration to a region of lower one. The rate of diffusion is affected by the concentration gradient, the reaction interface area, the thickness between the reaction interface and the decalcifier, tissue permeability, and temperature. The outside physical factors, such as temperature, EDTA concentration, agitation, ultrasound, irradiation of microwave, etc., have been widely reported. By purely mechanical means, diffusion of the agents was enhanced inside the tissue, and decalcification was accelerated. To increase the reaction interface area and shorten the thickness between the reaction interface and the decalcifier, lots of experiments have been displayed in previous reports with sawing a large bone into small thin logs before decalcification. However, tissue internal chemical factors concerning the tissue's intrinsic permeability have been neglected to some extent. The internal chemical factors that influence diffusion inside the organic material, including hydrophobic elements (e.g., fat) or lipophilic groups of amino acids, and the electric charges around the chemical ion passages in tissue.

**Figure 8:**
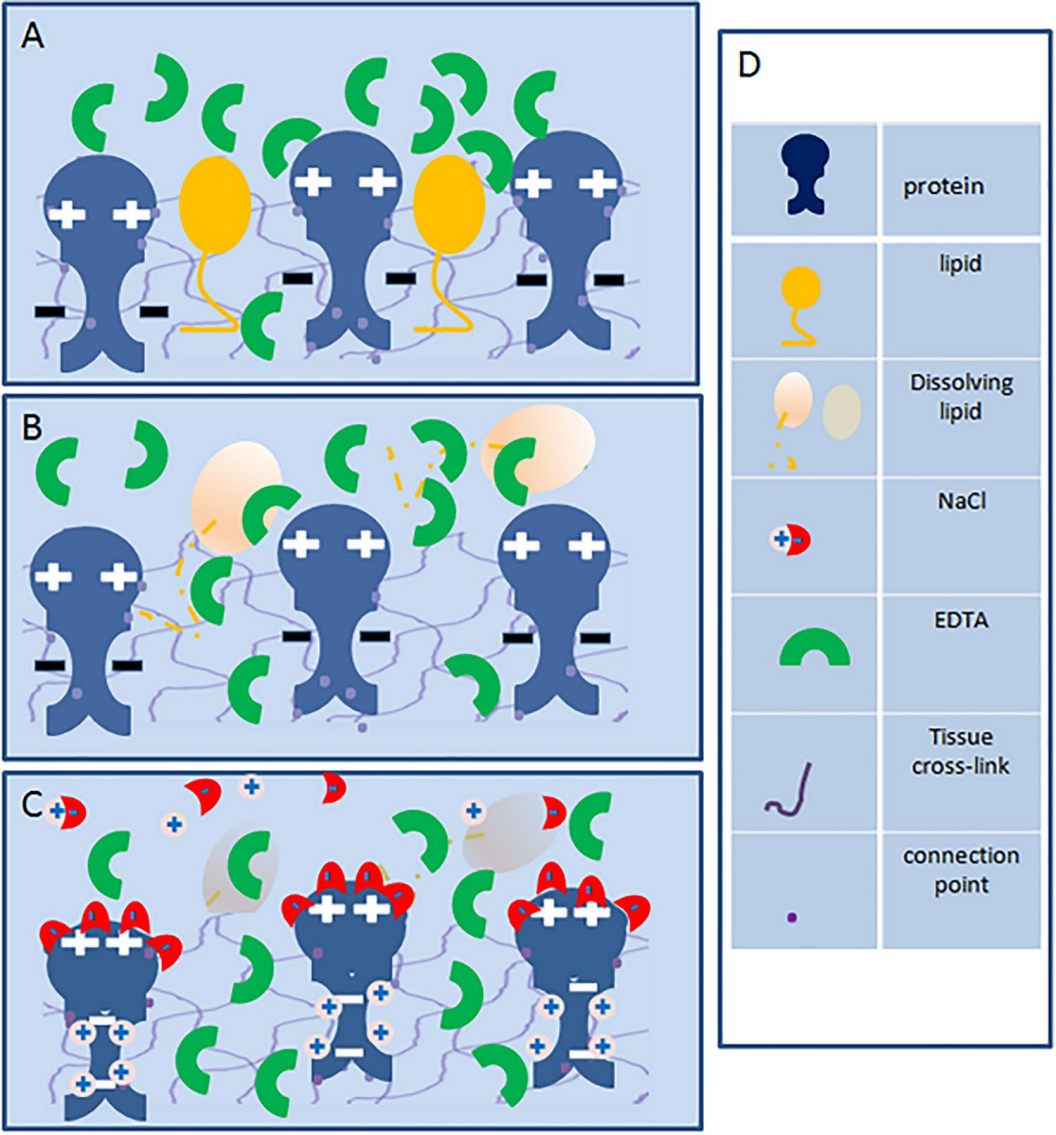
Fundamentals for accelerating diffusion of EDTA in tissue. (**A**) Proteins are cross-linked by induction of formalin-fixation and charged while contacted with an EDTA solution. Lipid droplets are inset amid the proteins. The charges and lipids impede EDTA penetration into the tissue. (**B**) EDTA penetration is enhanced after delipidation with detergents. (**C**) An additive of NaCl in the EDTA solution, shielding the charges around the penetration passages, accelerates further the tissue permeability of EDTA. (**D**) Symbol identifiers for (**A**–**C**).

Therefore, we hypothesize that the addition of hypertonic saline and detergents to the EDTA solution enhances the process of decalcification by accelerating tissue internal diffusion of relevant chemicals. The hydrophobic elements or lipophilic groups and the electric charges around the chemical ion passages in tissues might be removed and masked with detergents and hypertonic saline so that the polar molecules of the agents would penetrate the tissues with fewer disturbances, allowing for rapid permeation of the water soluble agent—EDTA (see Fig. 8). Thus, the concerning reagent transfer between the tissue to be decalcified and EDTA solution was facilitated when the detergents and hypertonic saline were present. Therefore, EDTA diffusion driven by the gradient concentration within the decalcified portion of tissue was accelerated, and decalcification became faster. The possible principle of this work is shown in Fig. 8.

For evaluation of the assumption, we determined the depth of EDTA infiltration in the tissue-mimicking gelatin (collagen) gel and the rate of decalcification in the ankle joints of mice. The tests on the collagen-rich gelatin gel have verified that detergents and hypertonic saline aided in the quick decalcification maybe by the speedy diffusion or permeation of EDTA. The higher the content of fat the gel contains, the more activity the detergent containing EDTA displays (Fig. 1). The assumption was further verified in the experiments of decalcification of the mouse paws (Fig. 2).

With the decalcification of the EDTA mixture of detergents and hypertonic saline, good morphological retention was demonstrated in the histological stains, TRAP, immunohistochemistry and in situ hybrid painting (Figs. 3, 4, 5, Figs. S8–S12), and mRNA was better preserved (Fig. 6) maybe due to the shorten decalcification. The outcomes via 26% EDTA-plus (45 °C) were equal or superior to those via the standard technique in tissue component retention (Fig. 7).

This is an important issue, especially in the field of surgical pathology, where minimal incubation duration, good preservation of morphology and target antigenicity, and good retention of nuclear acid integrity are always required. Thus the technique would benefit the laboratory by extending its application in urgent circumstances and increasing the staining qualities of the specimen. For example, in clinics, bone or bone marrow biopsies are always urgently needed for the diagnosis of hematologic cancer, metastatic tumors, and primary bone sarcoma. 

The chemicals used are cheap and easily accessed. The detergents and saline are routinely employed for delipidation and solution osmotic balance for preparation of histological stains, immunohistochemistry, and in situ hybrid in the laboratory. The procedure in the new decalcification way is simple and consistent with the diagnostic workflow currently used by pathologists. It has the potential to become a routine method for decalcification in the laboratory of histopathology.

For a successful application of the method, five universal points should be paid attention to, (1) Volume and concentration of decalcifying solutions. The higher concentration of the active agents will increase the rate at which calcium is removed from the bone to be decalcified. It must be remembered that the concentration of the active agent will be depleted as it combines with calcium so it is wise to use a large volume of the decalcifier or renew it several times during the decalcification process. (2) Temperature. Increased temperature will speed up the decalcification rate but will also increase the rate of tissue damage. Therefore, the temperature must be carefully controlled^39^. A working temperature of 30–45 °C is recommended, at which the decalcification would be accelerated in the EDTA mixture efficiently and histological and immunohistochemical analysis would not be affected. (3) Agitation. Strong agitation increases the rate by enhancing the active agent diffusion. (4) Fresh decalcifier. Freshly prepared decalcifying solutions have ready access to all surfaces of the specimen. This will enhance diffusion and penetration into the specimen and facilitate ionization and removal of calcium. (5) Decalcification duration. Using the 26% EDTA-plus at 45 °C, 24 h-decalcification worked well for adult mouse ankle joints. For the bone tissues derived from mice of a divergent age or other species of animals, optimization of decalcification should be performed for the duration.

Despite successfully speeding up the decalcification, at least three weaknesses should be paid attention to when using the current technique. (1) Application of this technique should be avoided in the tissues with an intention for other heavy metal detection (e.g., Fe, Cu, etc.) or the tissue block staining involving heavy metals (e.g., Fe in tissue block stain with Weigert Hematoxylin) since the method would extract both calcium and other heavy metals from the tissues or the dye of the stain. (2) The technique is not recommended for use in the tissue to be decalcified for lipid measurements or staining because the lipids in the tissue tend to redistribute or lose after detergent permeabilization of the decalcification solution. (3) Other salts or detergents, which may also increase the efficiency of decalcification, were not involved in the work and should be optimized for the best concentration and processing if applied.

## Materials and methods

This work, inclusive of tissue processing, cutting, staining, gel-relevant experiments, etc. was completed in a laboratory with a room temperature of 23 °C, except as stated.

### Gel preparation

4% gelatin gel (w/v) (Cat# 4274.1, Carl Roth, Germany) in distil water was prepared by heating using a microwave. After cooling to 55–60 °C, the melted gel was added with labeling chemicals (Weigert A/B mixture at a ratio of 1:1, Cat# X906.1 and X907.1, Carl Roth, Germany) in a volumetric ratio of 5:1. Take 3 ml of the melted labeling gel into a 15-ml tube, cast the gels in 4 °C for 30 min, and balance the tube at room temperature for 30 min before EDTA (Cat# CN06.4, Carl Roth, Germany) or the mixture addition (for more details, refer to Table S2). For the preparation of fat-contained gelatin gel, add _~_ 0.1 g of lipids extracted from a liver (to be described down) into a 15-ml melted labeling gel and mix well before distributing 3 ml of the gel to a tube and casting.

### Lipids extracted from liver

We used Folch’s extraction procedure^40^ for isolating lipids from a liver. It takes advantage of the biphasic solvent system consisting of chloroform/methanol/water in a volumetric ratio of 8:4:3. For more details, refer to Table S2. With the method, we yielded fats of 0.29 g from a liver weighing 1.34 g. The liver was derived from an 8-week-old male C57BL/6J mouse.

### Preparation of eggshell powder and decalcification

Bavaria cracked chicken eggshells from Germany were collected and washed. The dry-washed eggshells were crushed and ground in a Pulver (Cell crusher, Cat# 538,003, Kiskerbiotech GmbH and Co KG, Germany). After the powder was sieved with a sifter (Ø, 1 mm), the sieved pellets were washed with distil water and dried completely in an oven. Then a decalcification reaction was carried out by mixing 0.5 g of the chicken eggshell granule with 45 ml of 26% EDTA (pH = 7.4) or 26% EDTA mixtures (pH = 7.4). The 26% EDTA mixtures include 26% EDTA containing 5% Saline (26% EDTA + sa), 26% EDTA containing 0.5% Tween-20 (Cat# 9127.1, Carl Roth, Germany), and 1%Triton-X100 (Cat# 3051.3, Carl Roth, Germany) (26% EDTA + de), and 26% EDTA-plus (Fig. S7). The same volume of PBSx1 mixed with 0.5 g of eggshell granule served as a control. For more details, refer to Table S2. At the ends of 30 min, 1 h, and 3 h after incubation, the decalcification reaction under agitation was stopped to measure the weight of the remaining eggshell pellet.

### Animals and specimen preparations

This study was approved by the Regional Ethical Committee and the Government of Upper Bavaria, reference number: ROB-55.2–2532.Vet_02-19–69 and ROB-55.2–2532.Vet_02-16–115, based on a certified biostatistician's prior evaluation of animal study plan design and group sizes. All protocols regarding animal handling and experiments comply with Directive 2010/63/EU and the NIH Guidelines, and were reported in accordance with ARRIVE guidelines^41^.

Three cohorts of mice were used for this work and summarized in Table S1 on the strain, gender, age, body and hind paw weight, and source. These mice were euthanized in deep anaesthesia (Ketamin 150 mg/kg and Xylazin 15 mg/kg) at piloted time points and examined post-mortem for macro-anatomically pathological changes. The left or right hind paws were subsequently amputated at the level immediately above the external malleolus. The excised paws were weighed with a Sartorius fine balance (As-wägetechnik, Germany), skinned, and fixed in 4% (w/v) paraformaldehyde at 4 °C overnight. Decalcification was then performed under constant agitation for either subsequently piloted decalcification evaluation or the following paraffin embedding and histological/immunohistochemical stains (see the column of “purposes” in Table S1).

For the decalcification evaluation, 96 hind paws of male DBA/1 mice, 20 weeks in age, were randomly distributed to 11 groups (see Table S1) with 3—10 paws each. These paws were immersed in a volume of a solution of 200 times the samples (e.g., 1 g in 200 ml) for decalcification.

To evaluate the effects of the additives on decalcification of 15% EDTA at 23 °C, the solutions (see Table S1) prepared are 15% (w/v, pH = 7.4) EDTA, 15% EDTA containing 5% (w/v) NaCl (15% EDTA + sa), 15% EDTA containing 1% (v/v) triton-X100 and 0.5% (v/v) tween-20 (15% EDTA + de), 15% EDTA containing 5% saline, 1% triton-X100 and 0.5% tween-20 (15% EDTA-plus), and 5% saline containing 1% triton-X100 and 0.5% tween-20 (saline + deter). The decalcification with incubation in the solutions at room temperature (23°C) was stopped at the ends of 6 h, 24 h, and 3 days after the incubation. Three or four samples for each decalcification solution and time point were taken out for macro alizarin red staining to measure the remaining mineral of the distal portion of the tibia after decalcification. As controls, three samples in “15% EDTA RT” and “saline + deter” groups were picked out before decalcification and 24 h after the decalcification, respectively, for the macro alizarin red staining (refer to Fig. 2, Tables S1, S4). To assess the decalcification efficiency at a higher temperature we repeated the experiment for decalcification at 45 °C (Table S1 and Fig. 2).

In the same way, we also prepared 26% (w/v) EDTA, and 26% EDTA containing 5% saline, 1% triton-X100, and 0.5% tween-20 (26% EDTA-plus) and decalcified the samples at 23 °C or 45 °C to assess the decalcification efficiency (refer to Table S1 & Fig. 2) in a higher concentration of EDTA and its mixtures.

For assessment of morphological details following the decalcification, 12 right hind paws of C57 BL76J male mice, ranging 8.5—10 weeks in age, were randomly divided into two groups with 6 paws each (see Table S1). For the estimation of antigenicity preservation (see Table S1) after decalcification, we selected 12 left hind paws over 0.18 g in weight, from 20-week-old DBA/1 mice which had been immunized and boosted with bovine collagen II and human fibrinogen^36^. The 12 immunized paws were randomly and equally divided into two groups. One group objected to the decalcification of 26% EDTA-plus (45 °C) for 24 h (see Table S1). As controls, the other group was decalcified with the standard technique—15% EDTA (RT) for 1 week^35^. Following the decalcification, the samples were ready for paraffin embedding and sectioning.

### Paraffin embedding, sectioning, and collection

Following decalcification, tissues were trimmed, washed in running tap water, and soaked in deionized water for 30 min each. Then the specimens were processed for dehydration in ethanol and clearing in xylol, before infiltration and embedding in molten paraffin wax at 65 °C. For more details, refer to Table S3. For embedding, the samples were oriented with the external malleolus down for the right paw or the internal malleolus down for the left paw so that the soles of the paws were perpendicular to the cutting face.

After paraffin embedding, sagittal sections were cut at 5 μm with a Slee Cut 5062 rotary microtome (Slee Medical GmbH, Nieder-Olm, Germany). Paraffin ribbons at the middle of the ankle and tarsal joints were flattened in a water bath at 40 °C and collected onto polylysine microscope slides (Thermo Scientific) before drying at 45 °C overnight. For more details, refer to Fig. S2 in the Supplementary information. The paraffin sections were ready for histological staining.

### Histological staining

Before staining, paraffin sections from C57 BL/6J and immunized DBA/1 mice were deparaffinized with xylene (2 times for 5 min at RT), followed by rehydration through a graded series of 100%, 96%, and 70% ethanol, and finally with distilled water (each 3 min at RT). Morphological preservation was evaluated by hematoxylin (Harris hematoxylin, Cat# HHS80, Sigma) and eosin (HE), Safranin O (Saf O, Cat# T129.1, Carl Roth, Germany), toluidine blue (TB, Cat# 198,161-5G, Sigma), Sirius red, TRAP, and 4, 6-diamidino-2-phenylindole (DAPI, Mounting medium with DAPI, Cat# H-1200, Vector Lab.) staining, in situ hybrid painting (WCP probe for mouse Chromosome Y, Cat# FPRPR0168, ASI-Applied Spectral Imaging), and RNAScope in the sections of C57 BL/6J mice. Tissue antigenicity retention was assessed with immunostaining in the sections of immunized DBA/1 mice. The stains mentioned are the most frequently used method in histological laboratories. For the staining procedures in more detail, see Table S4 in Supplementary information.

### Immunohistochemical staining (IHC)

IHC was performed according to an established protocol^42,43^. Briefly, after deparaffinizing and rehydration, the sections were quenched in 1% hydrogen peroxide for 20 min and then submerged in 10 *mM* citrate buffer (pH 6.0) containing 0.05% tween-20 for 10 min at 85 °C and followed by cooling down to RT for 30 min for antigen retrieval. A blocking mixture of 2% mouse serum and 1% BSA was applied for 20 min to block the nonspecific binding. The sections were then incubated with preliminary antibodies (Table 2) for 30 min after tipping off the blocking solution, washed in PBS, and incubated with a biotin-conjugated anti-rat IgG antibody for 30 min. Following washing in PBS, incubation with streptavidin-conjugated HRP for 20 min was performed. Colorization was developed using 3,3-diaminobenzidine tetrahydrochloride (liquid DAB + Substrate DAKO). These sections were counterstained in Harris hematoxylin and examined under a light microscope (Carl Zeiss AG, Oberkochen, Germany).

**Table 2:** The preliminary antibodies used for IHC.

Antibodies	Epitopes	Targets	Working conc	Cat#	Sources
Rat anti-mouse Macrophage	MAC-2	Macrophage	1:50	H9939	Accurate chemical
Rat anti-mouse CD3	Glycoprotein CD3	T-cell	1:50	MCA500G	AbD Serotech
Rat anti-mouse CD45	Tyrosine-protein phosphatase C	Leukocyte	1:30	550,539	BD Pharmingen

For the validation of the experiments, a parallel experiment on the sections of the spleen, which served as positive or blank controls, was carried out.

### RNAscope

RNAscope was performed according to the manufacturer’s protocol with minor modifications. The paraffin sections were deparaffinized and rehydrated as described above. The RNAscope pretreatment included incubation in 3% hydrogen peroxide for 10 min at room temperature, tissue retrieval by boiling for 15 min in RNAscope Target Retrieval Reagent solution to undo the cross-linking and treatment with RNAscope Protease Plus at 40 °C for 20 min. After the pretreatment, the sections were hybridized with RNAscope Probe targeting mouse Actin (Cat # 316,741, ACD, Farmington, UT, USA) at 40 °C for 2 h. The signals were amplified, detected with RNAscope 2.5 HD assay-RED kit (Cat # 322,360, ACD, Farmington, UT, USA), and counterstained with DAPI mounting medium, where the fast red can be visualized in the red fluorescent channel under a Zeiss microscope described down. The RNAscope negative control probe-DapB (cat. no. 310043, ACD, Farmington, UT, USA) was used as the negative control.

### Alizarin gross staining

The decalcification degree was assessed by Alizarin stain at each time point (see Fig. 2) to determine the amount of mineral remaining with each decalcification method. After post-fixation in 90% ethanol and acetone, the samples were subjected to Alizarin staining until the bones became purple. Then the stained samples were ready for photographing followed by immersing in 50% glycerol prepared in distil water (for more details, see Table S4).

### Image acquisition and histological analysis

Images were acquired under the bright field illumination on a Zeiss upright microscope and imaging system, and recorded with a 2560 × 1920 pixel resolution and JPEG mode. For fluorescence-stained sections, blue and red fluorescence signals were inspected and photographed in DAPI and Cy-3 fluorescence channels, respectively (DAPI: 359 nm excitation and 457 nm emission; Cy-3: 546 nm excitation and 568 nm emission).

With the 40 × objective lens, HE, DAPI staining, in situ hybrid painting, and RNAscope were inspected and photographed. The HE-stained sections were examined for shrinkageand pyknotic cells. The DAPI-labeled nucleus area was determined by image analysis (Adobe Photoshop V5), and the total nuclei and the painting-stained nuclei were counted. The average nuclear area per nucleus (µm^2^/nucleus) was determined by the ratio between the total DAPI staining area and the total nuclear number. The painting’s positive nucleus rate was the ratio of the stained number to the total nucleus number. The positive signal integrated intensity of RNAscope was measured with ImageJ.

Using the 20 × objective lens, the positive cells and the areas involved were counted in the immunohistochemical stained sections and determined by the Image analysis mentioned^42,43^.

Under the 10 × objective lens of the microscope, the targeted areas in Sirius red, TRAP stained, and Saf O and TB stained sections were acquired and measured with image analysis.

By the 2.5 × objective lens, the Alizarin-stained samples were examined and focused on the region of tibia and then imaged at both low (5 µs) and high (500 µs) exposures for each sample. The high-exposure images were used to determine the amount of mineral remaining reflected by Alizarin staining (Fig. 2), and the low-exposure images the whole outline area of the samples. The relative area was calculated by the ratio of the mineral remaining area to the outline area. The images were acquired under a lighting condition except for the exposure time mentioned above and focusing on each new visual field. The lighting condition includes color cold, 0.3; color saturation,—0.2; light strength,—0.24; and color contrast, -0.48.

Qualitative assessment of mineral retention after decalcification was undertaken with macro-Alizarin stain, allowing for determination of the mineral loss at time points for each solution. The mineral loss in the relative area is 100% minus the relative area mentioned above.

In addition, the gelatin gel running in the tube for penetration speed detection was macroscopically registered with a Canon camera. The penetration depth from up down was measured and calculated as described in Fig. S1 at each time point.

### Data analysis

Data were presented as means ± SD. The software SPSS (IBM Corp. IBM SPSS Statistics for Windows, Version 11.0., USA) was employed for the analysis of correlation, Independent Samples T-test, one-way ANOVA, One-way ANOVA on ranks, or Paired Samples T-tests. One-way ANOVA on ranks was mainly used to identify differences in EDTA penetration in gel (for more details, see Method S1 in Supplementary Information) and EDTA efficiency of decalcification in tibias. A p < 0.05 was considered statistical significance.

### Supplementary Information

Supplementary Information.

## Data Availability

All relevant data are within the manuscript and its Supplementary information files.
