# Supplementary material for: Hypertonic saline- and detergent-accelerated EDTA-based decalcification better preserves mRNA of bones
Source: Sci Rep. 2024 May 13;14:10888. doi: 10.1038/s41598-024-61459-8 (PMC11091162; doi:10.1038/s41598-024-61459-8)
Supplement: Supplementary file 1 — Supplementary Information. [file 41598_2024_61459_MOESM1_ESM.docx]

Hypertonic saline- and detergent-accelerated EDTA-based decalcification better preserves mRNA of bones

Running title: additives accelerate EDTA-based decalcification

Zhongmin Li^1*^, Clara Wenhart^1^, Andreas Reimann^1^, Yi-Li Cho^1^, Kristin Adler^1^ & Goetz Muench^1^

^1^Advancecor GmbH, 82152 Martinsried, Germany.

***Corresponding author**

Zhongmin Li, Advancecor GmbH, Lochhamerstr. 29 A, 82152 Martinsried, Germany.

Tel: +49(0)89-2000204-13 Fax: +49(0)89-2000204-19 E-mail: li@advancecor.com

**Key Words:** Technique, Decalcification, EDTA, Histology, Delipidation, bone.

**Supplemental Material**

1. **Supplemental materials and methods**

**Table S1. Background of mice used for decalcification evaluation, histostains, and immunohistochemistry**

| **purposes** | **items** | **N** | **strains^1^** | **genders** | **means ± SD** | | |  |
| --- | --- | --- | --- | --- | --- | --- | --- | --- |
|  |  |  |  |  | **age(weeks)** | **body weight (g)** | **hind paw weight (g)^2^** |  |
| **decalci evaluation** | **15% EDTA/23°C** | **10** | **DBA/1** | **male** | **20 ± 0.00** | **24.5 ± 0.43** | **0.166 ± 0.008(L+R)** |  |
|  | **15% EDTA+saline/23°C** | **9** |  |  |  | **25.2 ± 0.82** | **0.165 ± 0.008(L+R)** |  |
|  | **15% EDTA+deter/23°C** | **9** |  |  |  | **26.5 ± 0.72** | **0.165 ± 0.007(L+R)** |  |
|  | **15% EDTA-plus/23°C** | **9** |  |  |  | **25.5 ± 0.36** | **0.164 ± 0.005(L)** |  |
|  | **saline+deter/23°C** | **3** |  |  |  | **26.8 ± 0.85** | **0.163 ± 0.007(L)** |  |
|  | **15% EDTA/45°C** | **9** |  |  |  | **23.7 ± 1.32** | **0.160 ± 0.006(L)** |  |
|  | **15% EDTA-plus/45°C** | **10** |  |  |  | **25.0 ± 0.98** | **0.161 ± 0.009(L+R)** |  |
|  | **26% EDTA/23°C** | **9** |  |  |  | **25.1 ± 0.97** | **0.166 ± 0.004(L+R)** |  |
|  | **26% EDTA-plus/23°C** | **9** |  |  |  | **23.9 ± 0.41** | **0.161 ± 0.007(L)** |  |
|  | **26% EDTA/45°C** | **9** |  |  |  | **24.4 ± 0.77** | **0.162 ± 0.008(L)** |  |
|  | **26% EDTA-plus/45°C** | **10** |  |  |  | **25.8 ± 0.89** | **0.166 ± 0.007(L+R)** |  |
| **histostains** | **15% EDTA/23°C** | **6** | **C57 BL/6J** | **male** | **9.3 ± 0.25** | **23.5 ± 1.06** | **0.160 ± 0.006 (R)** |  |
|  | **26% EDTA-plus/45°C** | **6** |  |  | **9.5 ± 0.32** | **23.8 ± 0.79** | **0.162 ± 0.002 (R)** |  |
| **immunohistochemistry** | **15% EDTA /23°C** | **6** | **DBA/1** | **male** | **20 ± 0.00** | **27.8 ± 1.38** | **0.186 ± 0.006(L)** |  |
|  | **26% EDTA-plus/45°C** | **6** |  |  |  | **26.6 ± 1.27** | **0.192 ± 0.008(L)** |  |
| 1. The DBA/1 and C57BL/6J mice were purchased from Janvier (Janvier Labs, France) and Charles River Laboratory (Sulzfeld. Germany), respectively. | | | | | | | | |
| 2. The left and right hind paws were indicated with L and R, respectively. | | | | | | | | |

**Table S2. Procedures for gelatin gel preparation, lipid extraction, and eggshell preparation**

|  | Procedures |
| --- | --- |
| **4% gel preparation** | 1. 4% gelatin gel was prepared using a weight/volume solution. |
|  | 2. Measure out distil water and pour it into a beaker. |
|  | 3. Weigh out gelatin and add it to the beaker containing distil water. |
|  | 4. Allow the agent to sit in solution for a few minutes. |
|  | 5. Use a stir bar and stirring plate to rapidly mix the solution. |
|  | 6. Cover the mouth of the beaker with paraffin film. |
|  | 7. Heat the beaker in the microwave. |
|  | 8. Take it out and swirl to mix well every 30 seconds until all the agent was dissolved. |
|  | 9. Remove the beaker from the microwave and swirl gently. |
|  | 10. Cool the solution to 55-60°C. |
|  | 11. Add labeling chemicals (Weigert A/B: 1/1) to the melted gel in a volumetric ratio of 1:5. |
|  | 12. Swirl to mix well. |
|  | 13. Fill 3 ml of the melted gels into a 15-ml tube. |
|  | 14. Cast the gel in 4°C for 30 min and balance it at room temperature for 30 min. |
| **Preparation of lipids** | 1. Weigh out a liver and put it in a 50-ml tube. |
|  | 2. Prepare a volume of a mixture (chloroform/methanol: 2/1). |
|  | 3. Take the mixture of 20 times the tissue sample (e.g., 1 g in 20 ml) into the tube. |
|  | 3. Homogenize the liver in the solvent mixture with a homogenizer. |
|  | 4. Agitate the whole mixture for 15-20 min in an orbital shaker at room temperature. |
|  | 5. Centrifuge the homogenate (5000 rpm) for 10 min to recover the liquid phase. |
|  | 6. Transfer the solvent to a 50-ml tube. |
|  | 7. Dilute the solvent with 0.2 volume (4 ml for 20 ml) of water. |
|  | 8. Mix the diluted solvent with vortexing for some seconds. |
|  | 9. Centrifuge the mixture at a speed of 2000 rpm to separate the two phases. |
|  | 10. Remove the upper phase by siphoning. |
|  | 11. Aliquot the lower chloroform phase containing lipids into 3 beakers. |
|  | 12. Evaporate at room temperature. |
|  | 13. Weight out the lipids (about 0.1 grams in each beaker). |
| **Preparation of eggshell** | 1. Wash and collect 2 cracked eggshells. |
|  | 2. Dry them out completely by baking them in an oven at 60°C for 2 hrs. |
|  | 3. Crush and grind in a Pulver^1^. |
|  | 4. Sieve the powder with a sifter (Ø, 1 mm). |
|  | 5. Wash the sieved material in distill water 3 times to remove driftage and dusty substance. |
|  | 6. Drain the granules and dry them out completely by baking in the oven at 60°C for 2 hrs. |
|  | 7. Weigh 0.5 g of the granule in a tube. |
|  | 8. Fill the tube with 45 ml EDTA solution and mix well. |
|  | 9. Decalcificate under agitation. |
|  | 10. Centrifuge the mixture with 2000g for 10 min. |
|  | 11. Wash the pellet with H2O by centrifuging 3 times. |
|  | 12. Dry it out completely by baking in the oven at 60°C for 2 hrs. |
|  | 13. Weigh the dry pellet. |

1. A cell crusher, purchased from Kiskerbiotech GmbH and Co KG, Germany. Cat# 538003.

**Table S3. Paraffin embedding procedures.**

| steps | solutions | conc (%) | time (min) | temp (°C) |
| --- | --- | --- | --- | --- |
| 1 | ethanol | 95 | 60 | RT |
| 2 | ethanol | 100 | 40 | RT* |
| 3 | ethanol | 100 | 40 | RT* |
| 4 | ethanol | 100 | 40 | RT* |
| 5 | xylene | ­ | 40 | RT* |
| 6 | xylene | ­ | 40 | RT* |
| 7 | xylene | ­ | 40 | RT* |
| 8 | paraffin | ­ | 60 | 65 |
| 9 | paraffin | ­ | 60 | 65 |
| 9 | paraffin | ­ | 60 | 65 |
| 10 | paraffin | ­ | 60 | 65 |
| RT, room temperature. *, under sonication. | | | |  |

**Table S4. Histological staining procedures.** The procedures were applied after paraffin slides were deparaffinized and rehydrated through graded ethanol to distilled water. The solutions were prepared as follows.

| Stains | Procedures |  |
| --- | --- | --- |
| Macro Alizarin staining | 1. Wash in deionized water. |  |
|  | 2. Immerse in 90% ethanol overnight. |  |
|  | 3. Incubate in acetone for 6 hrs. |  |
|  | 4. Wash in 1% KOH for 2 hrs. |  |
|  | 5. Immerse in Alizarin solution [1] overnight. |  |
|  | 6. Rinse in 1% KOH for 2 hrs. |  |
|  | 7. Place in 50 glycerol. |  |
|  |  |  |
| HE staining | 1. Wash in deionized water. |  |
|  | 2. Immerse in Harris hematoxylin for 2 minutes. |  |
|  | 3. Wash in water. |  |
|  | 4. Blue in running tap water for 10 minutes. |  |
|  | 5. Immerse in eosin for 40 seconds. |  |
|  | 6. Rinse in distilled water for 15 seconds. |  |
|  | 7. Dehydrate sections with two 20-second washes in 100% ethanol. |  |
|  | 8. Place in xylene for 30 seconds. |  |
|  | 9. Mount in Kanadabalsam. |  |
|  |  |  |
| Safranin O staining | 1. Wash in deionized water. |  |
|  | 2. Incubate in 0.1% safranin O for 1 min. |  |
|  | 3. Rinse in 1% acetic acid for 15 seconds. |  |
|  | 4. Rinse in running tap water for 15 seconds. |  |
|  | 5. Dehydrate sections with two 20-second washes in 100% ethanol. |  |
|  | 6. Place in xylene for 30 seconds. |  |
|  | 7. Mount in Kanadabalsam. |  |
|  |  |  |
| TB staining | 1. Wash in deionized water. |  |
|  | 2. Incubate in TB working solution (0.1% (w/v))[2] for 20 seconds. |  |
|  | 3. Rinse in distilled water for 15 seconds. |  |
|  | 4. Dehydrate in three changes of 100% ethanol. |  |
|  | 5. Place in xylene for 2 min. |  |
|  | 6. Mount in Kanadabalsam. |  |
|  |  |  |
| TRAP staining | 1. Wash in deionized water. |  |
|  | 2. Place slides in pre-warmed TRAP staining solution [3] (37°C). |  |
|  | 3. Incubate at 37 °C for 30 mins. |  |
|  | 4. Rinse in distilled water. |  |
|  | 5. Counterstain with 0.02% Fast Green for seconds. |  |
|  | 6. Rinse quickly in distilled water. |  |
|  | 7. Dehydrate in three changes of 100% ethanol. |  |
|  | 8. Place in xylene for 2 min. |  |
|  | 9. Mount in Kanadabalsam. |  |
| DAPI fluorescence staining | 1. Wash in deionized water. |  |
|  | 2. Incubate in 4% paraformaldehyde (prepared in 0.5% triton) for 10 mins. |  |
|  | 3. Rinse in 0.5% triton prepared in 1xPBS for 15 seconds. |  |
|  | 4. Mount in DAPI contained anti-fade mounting medium. |  |
|  | Sirius Red staining | 1. Stain in Sirius red solution [4] for one hour. |
|  |  | 2. Wash briefly in distilled water. |
|  |  | 3. Physically remove most of the water from the slides by vigorous shaking. |
|  |  | 4. Dehydrate in three changes of 100% ethanol. |
|  |  | 5. Clear in xylene and mount in Kanadabalsam. |
|  |  |  |

Solutions

| 1. Alizarin Red Solution | Dissolve 1 mg of alizarin red S in 100 ml of 1% KOH |
| --- | --- |
| 2. TB stock solution (1%) | 1% TB prepared in 0.1% natriumtetraborate in water. Filtrate before use. |
| 3. TRAP staining solution | 1. Prepare Tartaric buffer by mixing 1.4 ml acetic acid, 4.6 g sodium acetate, |
|  | 5.7g tartaric acid and 457 ml H2O and adjust pH to 4.7 - 5.0. |
|  | 2. Prepare Naphthol ASMX Phosphate substrate by mixing 400 mg. |
|  | Naphthol AS-MX phosphate and 2 ml ethylene glycerol monoethylether. |
|  | 3. Prepare a staining solution by mixing 200 ml tartaric buffer, 1 ml naphthol |
|  | substrate, and 120 mg fast red violet LB salt. |
| 4. Sirius Red Solution | Dissolve 0.5 g of Sirius red F3B in 500 ml of a saturated aqueous solution of  picric acid and mix well. |

The relevant reagents were purchased from Sigma.

**Table S5. In situ hybrid painting procedures.**

| In situ hybrid | 1. Put the slides in 2XSSC(diluted with 20X) [1] at RT for 2 min. |
| --- | --- |
|  | 2. Dehydrate in ethanol series: ice cold 70%, 80%, and 100%, 2 min each. |
|  | 3. Air dry at RT for 30 min. |
|  | 4. Dip slides in denaturation solution [2] of 72 °C for 2 times. |
|  | 5. Apply 10 µl of the probe mixature [3]. |
|  | 6. Put a cover slip (18X18mm) and seal with rubber cement. |
|  | 7. Denature sample and probe together on a hot plate at 74 °C for 4 minutes. |
|  | 8. Place in an incubator or baking oven set at 37 °C for 12-16 hours. |
|  | 9. Remove the rubber cement and the coverslip. |
|  | 10. Wash the slides in 0.4XSSC [4] at 74 °C for 3-5 min. |
|  | 11. Dip slides in 4XSSC/ 0.1% Tween 20 [5] for 2 minutes. |
|  | 12. Mount with DAPI mounting medium. |

**Solutions**

| 1. 20XSSC | 1. Dissolve 87.6 g NaCl and 44.1 g Sodium Citrate in 500 ml of H2O. . |
| --- | --- |
|  | 2. Adjust pH to 7.4. |
| 2. Denaturation solution | 1. Mix 35 ml formamide, 10 ml distilled H2O, and 5 ml 20xSSC. |
| (70% formamide /2SSC) | 2. Adjust pH to 7.0 using HCL, heat to 72 °C. |
| 3. Probe mixature | Mix 8 µl probe and 2 µl denaturation solution |
| 4. 0.4 X SSC solutions | 1. Mix 1ml 20X SSC and 49 ml distilled water. |
|  | 2. Mix well and heat to 74 °C. |
| 5. 4 X SSC/0.1%Tween | Mix 100 ml 20X SSC, 400ml distilled water and 0.5 ml Tween 20. |

**
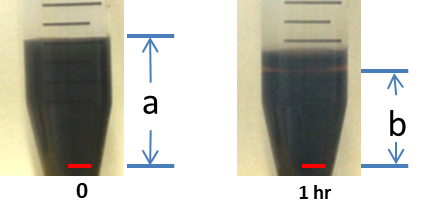
**

**Figure S1. Measurement of EDTA penetration depth in gel.** 1). Put a casted-gel-containing tube in a tube frame and maintain an upright position during the experiment. 2). Mark a site as a fixed position for each measurement at piloted time points, indicative of red color in the figure. The site is outside of the tube wall, 1 cm above the bottom (tip). 3). Photograph the tube with a rule alongside, immediately after the addition of EDTA solution (timepoint 0 in the figure), and at the piloted time points after the addition (e.g., 1 hr in the figure). 4). Measure the height of the gel from the fixed position to the top (indicative of “a“ in the figure) or the bottom of the yellow band (indicative of “b“ in the figure). 5). Determine the penetration depth from up down by the formula - “the penetration depth = a – b”.

**
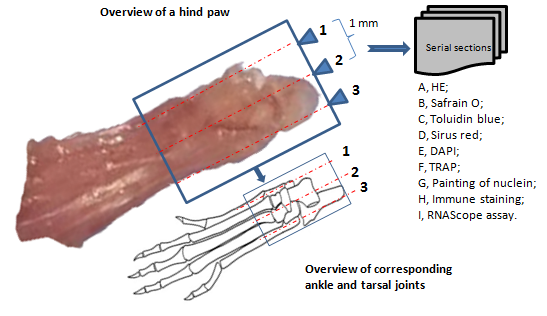
**

**Figure S2. Sagittal sections and staining in ankle and tarsal joints.** Following the decalcification of the hind paws of mice, various stains in serial middle sagittal sections, indicated by the numeral “2”, were used for histopathological evaluation. The region of interest for cutting in joints is indicated by the blue rectangles and the dashed red lines reflect the positions of the cutting planes.

**Method S1. One-way ANOVA on ranks**

One-way ANOVA on ranks is a nonparametric statistic, where ranks of the combined data instead of the nonnormally distributed numerical values, are used to compare with a standard one-way ANOVA.

This statistic gives a rough indication of differences among different treatments and the p-value is not exact. However, it is relatively simple and sufficient for the comparison effects of different groups. The steps were taken as follows.

1). Rank the original data by time point. The data of heterogeneity are 'tamed' by the ranking procedure, so the violations of standard ANOVA assumptions are not so serious.

2). The ranked data that was extracted were employed for comparison. To sharpen the difference among the groups, please note that the non-interventional groups or time points (or the non-intervention data, e.g., observations measured at “0” of time points, and PBS or saline control group) were excluded from the analysis.

3). One-way ANOVA was conducted that examined the difference effects, based on the ranked data.

**2. Supplemental results**

**Figure S3. Principle for alternation of Weigert hematoxylin in color by EDTA.** Weigert hematoxylin is a nuclear staining agent that is used in many non-routine techniques (special stains). It contains hematoxylin (Weigert A) and ferric chloride (Weigert B). Weigert A appears brown, and Weigert B is colorless and transparent. The mixture of Weigert A and B turns black when the hematoxylin forms a complex with ferric ion (Weiger A+B). When the complex contacts EDTA, the metal ion is chelated, leaving the free hematoxylin, and the uncomplexed hematoxylin returns to its brown color (Weigert A+B+EDTA). As a control, the complex remains unchanged in color when it contacts NaCl or detergents (Weigert A+B+sa/de).


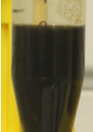

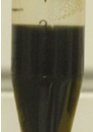

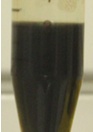

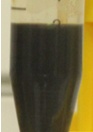

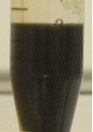

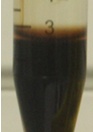

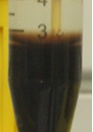

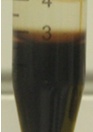

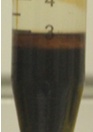

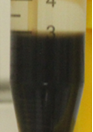

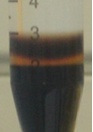

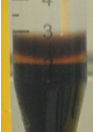

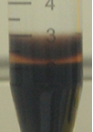

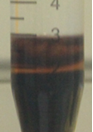

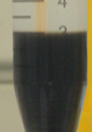

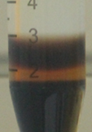

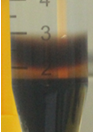

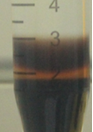

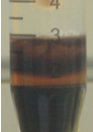

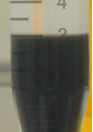

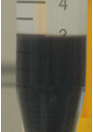

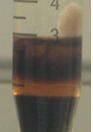

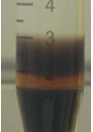

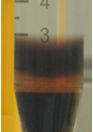

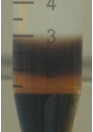


**15% EDTA**

**EDTA+0.9% sa**

**EDTA+5% sa**

**EDTA+10% sa**

**PBSx1**

**0**

**15‘**

**2 hrs**

**4 hrs**

**6 hrs**

A

**
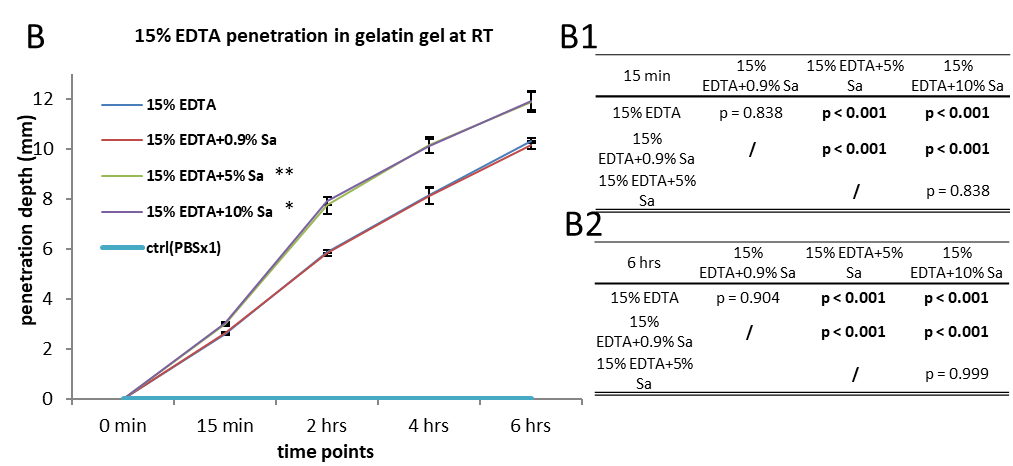
**

**Figure S4. EDTA penetration varied with addition of saline.** (A). The EDTA (15%, w/v) penetration depth varied with the addition of 0 (15%EDTA), 0.9% (EDTA+0.9% sa), 5% (EDTA+5% sa), and 10% NaCl (EDTA+10% sa). The saline mixtures were prepared in 15% EDTA. PBSx1 served as a control. (B). The penetration depth was measured in triplicate at the piloted time points, and comparison of the average values among the experimental groups within the period 15` - 6hrs, with one-way ANOVA on ranks and Tukey HSD, resulted in statistically significant difference; *, for all, p < 0.001, vs 15% EDTA, or 15% EDTA+0.9% sa; **, for all, p < 0.001*,* vs 15% EDTA, or 15% EDTA+0.9% sa; N = 4 (4 average values for 4-time points per group). At the first (15 min) and final (6 hrs) time points after the addition of EDTA, the penetration depths (N = 3 per group) were compared among the experimental groups, with one-way ANOVA and Tukey HSD. The p values of the pairwise comparisons were given in Tables B1 (15 min) and B2 (6 hrs). Statistically significant differences were marked in boldface in the tables. The results indicate that the addition of hypertonic saline to the EDTA accelerated the penetration rate. It was evident that 15% EDTA+5% sa and 15% EDTA+10% sa had nearly equal effects on the penetration. Green bars in (A) indicate 10 mm.


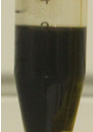

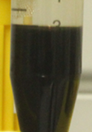

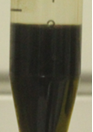

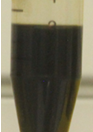

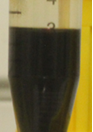

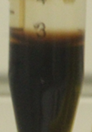

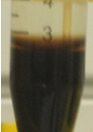

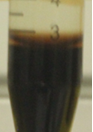

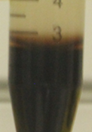

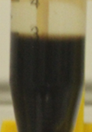

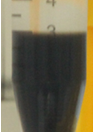

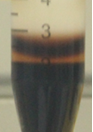

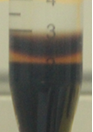

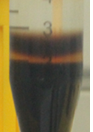

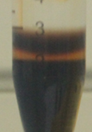

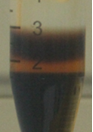

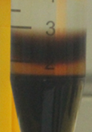

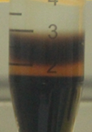

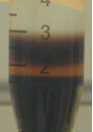

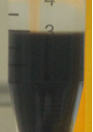

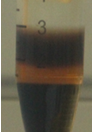

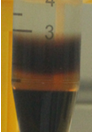

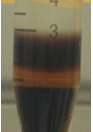

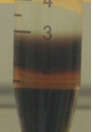

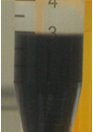


**15% EDTA**

**EDTA+0.02%Tw+0.04%Tr**

**EDTA+0.1%Tw+0.2%Tr**

**EDTA+0.5%Tw+1%Tr**

**PBSx1**

**0**

**15‘**

**2 hrs**

**4 hrs**

**6 hrs**

A

**
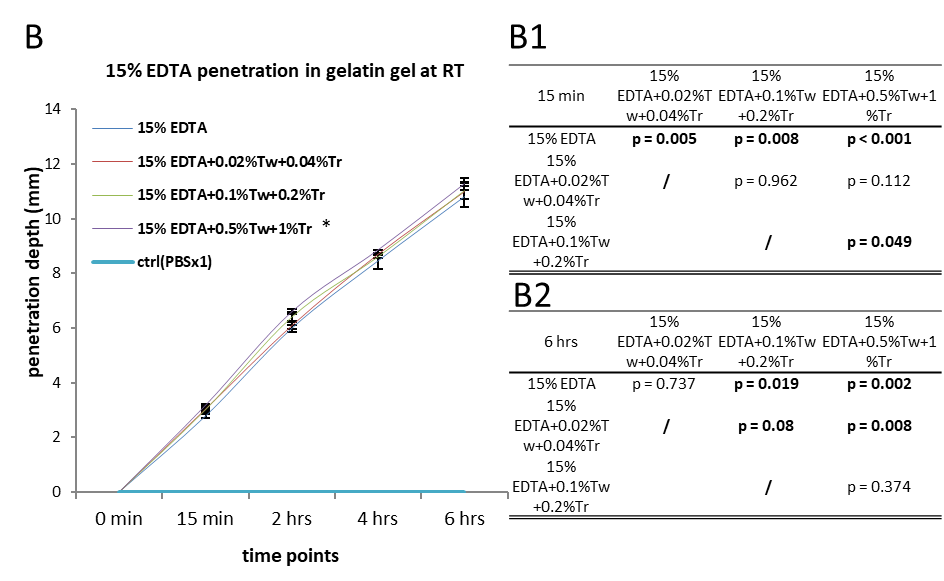
**

**Figure S5. EDTA penetration varied with addition of detergents**. (A). The EDTA (15%, w/v) penetration depth varied with the addition of 0 (15% EDTA); 0.02% Tween 20, and 0.04% Triton X100 (EDTA+0.02%Tw+0.04%Tr); 0.1% Tween 20 and 0.2% Triton X100 (EDTA+0.1%Tw+0.2%Tr); and 0.5% Tween 20 and 1% Triton X100 (EDTA+0.5%Tw+1%Tr). The detergent mixtures were prepared in 15% EDTA. PBSx1 served as a control. (B). The penetration depth was measured in triplicate at each time point. Comparison of the average values measured among the experimental groups within the period 15`- 6 hrs, with one-way ANOVA on ranks and Tukey HSD, resulted in a statistically significant difference; *, for all, p < 0.001, vs 15% EDTA, 15% EDTA+0.02%Tw+0.04%Tr, or 15% EDTA+0.1%Tw+0.2%Tr, N = 4 (4 average values for 4 time points per group). At the first (15 min) and final (6 hrs) time points after the addition of EDTA, the penetration depths (N = 3 per group) were compared among the experimental groups, with one-way ANOVA and Tukey HSD. The p values of the pairwise comparisons were given in Tables B1 (15 min) and B2 (6 hrs). In the tables, statistically significant differences were marked in boldface. The result indicates that the addition of 0.5% Tween and 1% Triton to the EDTA exerts the strongest effect on the penetration among the groups. Green bars in (A) indicate 10 mm.

**Figure S6. EDTA penetration varied with addition of saline and detergents**. The penetration depth was measured in triplicate at the piloted time points. Of note, the EDTA penetration depth varied with the addition of saline (15% EDTA+sa, 15% EDTA+5% sa), detergents (15% EDTA+de, 15% EDTA+0.5%tw+1%tr), or the mixture - 15% EDTA-plus (15% EDTA+5%sa+0.5%tw/1%tr). The mixtures were prepared in 15% EDTA. PBSx1 served as a control. Comparison of the average values measured among the experimental groups within the period 15` - 6hrs, with one-way ANOVA on ranks and Tukey HSD, resulted in a statistically significant difference; *, for all, p < 0.002, vs 15% EDTA, 15% EDTA+sa, or 15% EDTA+de, N = 4 (4 average values for 4 time points per group). At the first (15 min) and final (6 hrs) time points after the addition of EDTA, the penetration depths (N = 3 per group) were compared among the experimental groups, with one-way ANOVA and Tukey HSD. The p values of the pairwise comparisons were given in Tables B (15 min) and C (6 hrs). In the tables, statistically significant differences were marked in boldface. The result indicates that 15% EDTA-plus (addition of 5% sa, 0.5% Tween 20 and 1% Triton to the EDTA) has the strongest effect on the penetration rate among the groups.

**Figure S7. Eggshell weight loss in 26% EDTA and its mixtures at different time points under room temperature (RT).** The EDTA mixtures, including 5% NaCl (26% EDTA+sa), 0.5%Tween/1%trion (26% EDTA+de), and 5% NaCl and 0.5%Tween/1%trion (26% EDTA-plus), were prepared in 26% EDTA. PBSx1 served as a control. The eggshell weight was measured and the eggshell weight in loss was calculated in quadruplicate at each time point. Comparison of the average values of the weight loss among the experimental groups, with one-way ANOVA on ranks and Tukey’s HSD, resulted in a statistically non-significant difference. For all, p > 0.78; N = 3 (3 average values for 3 time points per group).


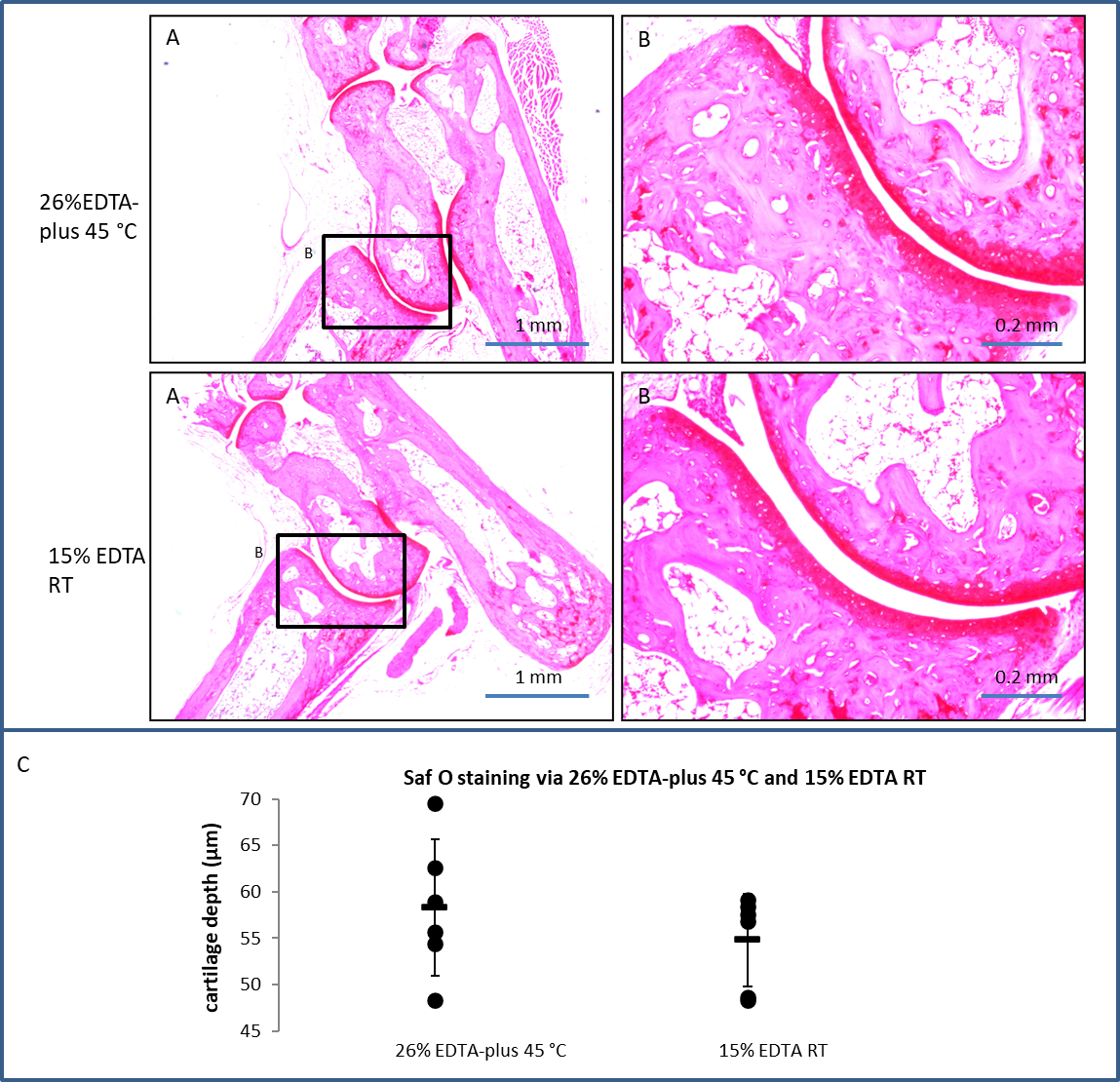


**Figure S8. Saf O staining in tissues following decalcification with either 26% EDTA-plus 45 °C or 15% EDTA RT.** The regions indicated by black rectangles in (A) were magnified in the corresponding images in (B). (C). In terms of the cartilage depth of the tibia distal portion, the comparison between 26% EDTA-plus 45 °C and 15% EDTA RT, with Independent Samples T test, resulted in a statistically non-significant difference (N = 6 pairs, p = 0.53).


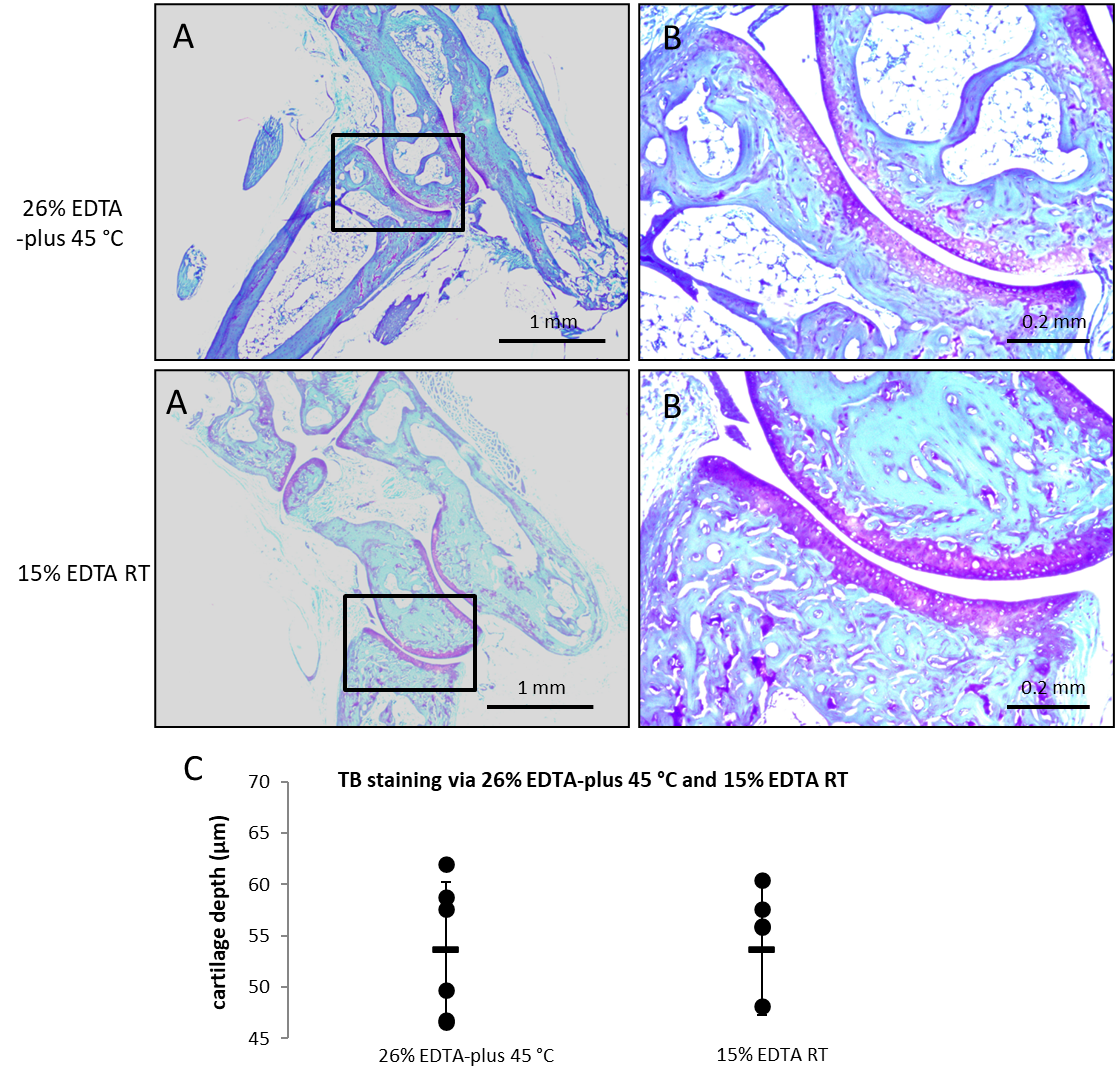


**Figure S9. TB staining in tissues following decalcification with either 26% EDTA-plus (45 °C) or 15% EDTA (RT).** The regions indicated by black rectangles in (A) were magnified in the corresponding images in (B). (C). In terms of the cartilage width of the tibia distal portion, the comparison between EDTA-plus and 15% EDTA, with Independent Samples T-test, resulted in a statistically non-significant difference (N = 6 pairs, p = 0.59).


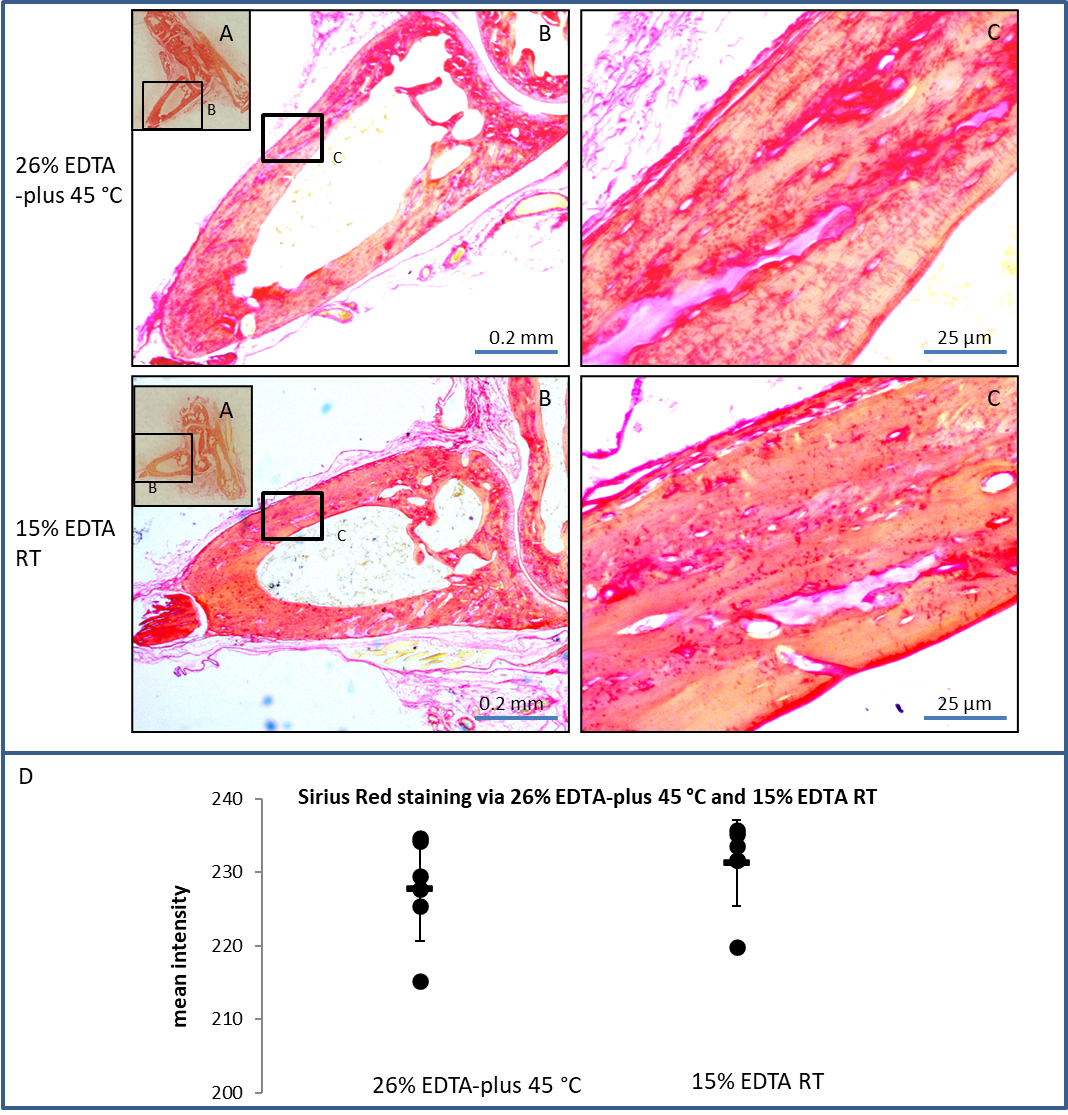


**Figure S10. Sirius Red staining in tissues following decalcification with either 26% EDTA-plus (45 °C) or 15% EDTA (RT).** The regions indicated by black rectangles in (A) and (B) were magnified in the corresponding images of (B) and (C), respectively. The mean intensity of red in the middle shaft of the tibia via decalcification of either 26% EDTA-plus (45 °C) or 15% EDTA (RT), as indicated in (C), was measured and compared in (D). The comparison between the two groups, with Independent Samples T test, resulted in a statistically non-significant difference (N = 6 pairs, p = 0.07).

**
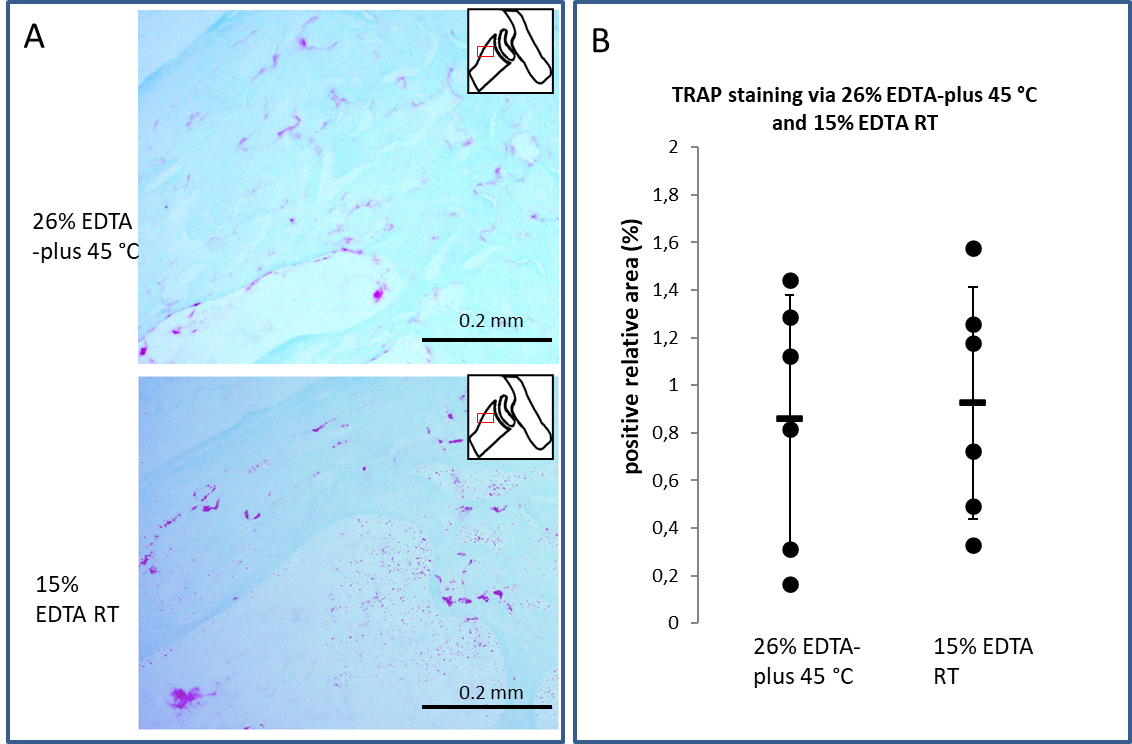
**

**Figure S11. TRAP staining in tissues following decalcification with either 26% EDTA-plus (45 °C) or 15% EDTA (RT).** (A). The regions indicated by red rectangles were magnified in the corresponding images. (B). In terms of the positive relative area of the tibia distal portion, the comparison between 26% EDTA-plus 45 °C and 15% EDTA RT groups, with Independent Samples T-test, resulted in a statistically non-significant difference (N = 6 pairs, p = 0.91).


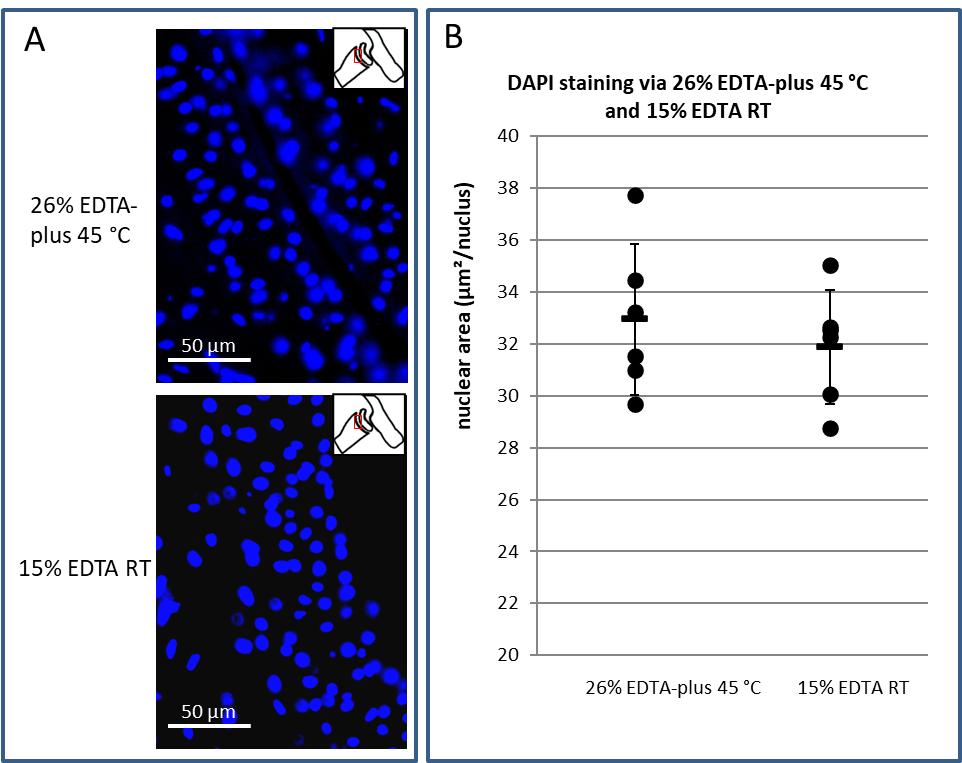


**Figure S12. DAPI staining in tissues following decalcification with either 26% EDTA-plus (45 °C) or 15% EDTA (RT).** (A). The regions indicated by red rectangles in insets were magnified in the corresponding images. (B). Nuclear area per cell was determined by image analysis and the comparison of the nuclear area per cell between 26% EDTA-plus and 15% EDTA groups, with Independent Samples T-test, resulted in a statistically non-significant difference (N = 6 pairs, p = 0.52).
